# Arginine-Based Poly(I:C)-Loaded Nanocomplexes for the Polarization of Macrophages Toward M1-Antitumoral Effectors

**DOI:** 10.3389/fimmu.2020.01412

**Published:** 2020-07-07

**Authors:** Tamara G. Dacoba, Clément Anfray, Francesco Mainini, Paola Allavena, María José Alonso, Fernando Torres Andón, José Crecente-Campo

**Affiliations:** ^1^Center for Research in Molecular Medicine and Chronic Diseases (CIMUS), IDIS Research Institute, Universidade de Santiago de Compostela, Santiago de Compostela, Spain; ^2^Department of Pharmacology, Pharmacy and Pharmaceutical Technology, School of Pharmacy, Universidade de Santiago de Compostela, Santiago de Compostela, Spain; ^3^Laboratory of Cellular Immunology, Humanitas Clinical and Research Center IRCCS, Milan, Italy

**Keywords:** poly(I:C), toll-like receptor (TLR) 3, tumor-associated macrophages (TAMs), arginine-rich peptides, nanocomplexes, cancer immunotherapy

## Abstract

**Background:** Tumor-associated macrophages (TAMs), with M2-like immunosuppressive profiles, are key players in the development and dissemination of tumors. Hence, the induction of M1 pro-inflammatory and anti-tumoral states is critical to fight against cancer cells. The activation of the endosomal toll-like receptor 3 by its agonist poly(I:C) has shown to efficiently drive this polarization process. Unfortunately, poly(I:C) presents significant systemic toxicity, and its clinical use is restricted to a local administration. Therefore, the objective of this work has been to facilitate the delivery of poly(I:C) to macrophages through the use of nanotechnology, that will ultimately drive their phenotype toward pro-inflammatory states.

**Methods:** Poly(I:C) was complexed to arginine-rich polypeptides, and then further enveloped with an anionic polymeric layer either by film hydration or incubation. Physicochemical characterization of the nanocomplexes was conducted by dynamic light scattering and transmission electron microscopy, and poly(I:C) association efficiency by gel electrophoresis. Primary human-derived macrophages were used as relevant *in vitro* cell model. Alamar Blue assay, ELISA, PCR and flow cytometry were used to determine macrophage viability, polarization, chemokine secretion and uptake of nanocomplexes. The cytotoxic activity of pre-treated macrophages against PANC-1 cancer cells was assessed by flow cytometry.

**Results:** The final poly(I:C) nanocomplexes presented sizes lower than 200 nm, with surface charges ranging from +40 to −20 mV, depending on the envelopment. They all presented high poly(I:C) loading values, from 12 to 50%, and great stability in cell culture media. *In vitro*, poly(I:C) nanocomplexes were highly taken up by macrophages, in comparison to the free molecule. Macrophage treatment with these nanocomplexes did not reduce their viability and efficiently stimulated the secretion of the T-cell recruiter chemokines CXCL10 and CCL5, of great importance for an effective anti-tumor immune response. Finally, poly(I:C) nanocomplexes significantly increased the ability of treated macrophages to directly kill cancer cells.

**Conclusion:** Overall, these enveloped poly(I:C) nanocomplexes might represent a therapeutic option to fight cancer through the induction of cytotoxic M1-polarized macrophages.

## Introduction

The discovery of the capacity of the immune system to fight and eliminate tumors has represented a major paradigmatic change in the treatment of cancer, classically addressed with cytotoxic drugs (1, 2). Despite the inherent anti-tumoral capacity of immunocompetent cells, tumors produce immunosuppressive signals that lead to tumor immune tolerance, thus facilitating tumor progression (3–5). Among the different cells involved in this process, tumor-associated macrophages (TAMs) are key players with the capacity to promote the proliferation of cancer cells, angiogenesis and metastasis (5–8). TAMs present anti-inflammatory and tolerogenic features, that are similar to M2-like macrophages (9). Importantly, recent investigations have proposed the possibility to reprogram TAMs toward pro-inflammatory and anti-tumoral M1 states as a promising approach to re-activate the immune response against tumors (8, 10–13).

An important strategy to re-educate TAMs toward M1-like phenotypes (14, 15), has relied on the use of agents that activate the toll-like receptors (TLRs) (13, 16). Upon interaction with their corresponding agonists, TLRs activate MyD88 and TRIF pathways, thereby triggering innate and adaptive immune responses (17, 18). Indeed, some of these agonists are already marketed, or under clinical trials, for vaccination and/or cancer applications (18). Among the different TLR agonists, poly(I:C), a double-stranded (ds)RNA that activates the TLR3, has shown the capacity to polarize TAMs toward M1-like anti-tumoral phenotypes (19). Nevertheless, the clinical potential of poly(I:C) has been undermined by its indiscriminate biodistribution, that leads to an unrestrained immune activation and systemic inflammation, with serious toxic effects (20–24). Another major issue for the use of poly(I:C) in the clinic is related to its systemic degradation (21). In this sense, the association of poly(I:C) into a nano-delivery system could protect the drug and improve its transport to the tumor site and, consequently, ameliorate its safety profile (21, 25–30).

Synthetic nanosystems for polynucleotide delivery are mainly based on their complexation with positively charged lipids or polymers (31–33). For example, it has been reported that the complexation of poly(I:C) with cationic polymers, i.e., polyethyleneimine (PEI), leads to positive *in vivo* results in different cancer models (34), and is currently in a phase I clinical trial (35). Unfortunately, PEI itself is not absent of systemic toxicity (36). In our research group, alternative nanocarriers for the delivery of polynucleotides have already been explored. Based on the known capacity of cell penetrating peptides (CPPs) to efficiently condense nucleic acids and facilitate their transport across biological barriers (37), we have developed polyarginine- (pArg) and protamine-based nanosystems, which have shown the capacity to efficiently deliver different polynucleotides (38–40). Indeed, we have recently reported the formation of nanocomplexes of polynucleotides with cationic molecules, and their posterior envelopment with an hydrophilic anionic polymer, named as enveloped nanocomplexes (ENCPs), as a way to facilitate the delivery of miRNA to the brain (40).

As a whole, despite the potential of poly(I:C) for polarizing macrophages toward an anti-tumoral M1-like phenotype with the capacity to fight tumors, the *in vivo* administration of this TLR3 agonist presents significant side effects. Therefore, here we aimed at engineering a nanocomplex to improve the capacity of poly(I:C) to polarize macrophages toward M1-like phenotypes. After an optimization process, we evaluated the *in vitro* capacity of the developed poly(I:C) nanocomplexes to polarize primary human monocyte-derived macrophages toward pro-inflammatory M1-like anti-tumoral phenotypes.

## Materials and Methods

### Materials

n-Butyl-poly(L-arginine) hydrochloride (pArg) (150 arginine residues, MW 24 kDa) and the different pegylated-poly(L-glutamic acid) (PEG–PGA) polymers were purchased from Polypeptide Therapeutic Solutions (PTS, Valencia, Spain). For the PEG–PGA, three types of branched conformations were acquired: PGA, either 10 or 30 units, with a molar substitution degree of 10 or 30% of PEG (5 kDa), referred as: PEG_5k_10–PGA10, PEG_5k_10–PGA30, and PEG_5k_30–PGA10. Also, two conformation of the diblock PEG-PGA were purchased: 10 units of PGA and a 20 kDa PEG tail; and 30 units of PGA with a 5 kDa PEG tail, named as diblock PEG_20k_-PGA10 and diblock PEG_5k_-PGA30, respectively.

Octa-D-arginine (r8) and laurate octa-D-arginine (C12r8) were obtained from ChinaPeptides (Shanghai, China). Sodium hyaluronate (HA) (MW 57 kDa) was purchased from Lifecore Biomedical (MN, USA). HMW poly(I:C) and HMW poly(I:C)-rhodamine were acquired from InvivoGen (CA, USA). Endotoxin-free water was used for all the *in vitro* experiments.

### Preparation of the Nanocomplexes

#### Screening of Arginine-Rich Polymers

To 400 μL of arginine-rich polymer solution (0.5, 1, or 2 mg/mL), 200 μL of poly(I:C) (at 1 or 0.5 mg/mL) were added under mild magnetic stirring. Weight ratios polymer to poly(I:C) 1:1, 2:1 and 4:1 were tested (Supplementary Table 1). After 1–5 min of stirring, the resulting nanocomplexes were allowed to stabilize for at least 3 min before further characterization or envelopment.

#### Envelopment With PEG–PGA Polymers

A volume of 400 μL of a PEG–PGA aqueous solution at 1 mg/mL was added to a round bottom flask, and the water was evaporated in a rotavapor (Heidolph Hei–VAP Advantage, Schwabach, Germany) for 10 min, at 37°C, under vacuum and mild rotary speed, until a thin film was formed. Then, the same volume of nanocomplexes (with a poly(I:C) concentration of 0.33 or 0.17 mg/mL) (Supplementary Table 2), was added to the round bottom flask, in order to achieve their envelopment by PEG–PGA. The same the same rotary speed was maintained for 10 min, at room temperature and atmospheric pressure.

#### Envelopment With HA

To 250 μL of the nanocomplexes with a poly(I:C) concentration of 0.33 or 0.17 mg/mL, the same volume of an HA solution of concentrations ranging from 0.25 to 2.00 mg/mL, was added under mild magnetic stirring, for a final poly(I:C) to HA weight ratio of 1:1.5, 1:3, or 1:6 (Supplementary Table 3). The ENCPs were allowed to be formed for 5 min under stirring, and to be stabilized during other 5 min prior to their characterization.

### Nanoparticle Characterization by Dynamic Light Scattering (DLS)

The mean particle size (Z-average) and polydispersity index (PDI) of the non-diluted samples were characterized by DLS. The zeta potential values were determined by Laser Doppler Anemometry (LDA), measuring the mean electrophoretic mobility after a 20-times dilution of the ENCPs in ultrapure water. These properties were measured using a Zetasizer® NanoZS, with the software Zetasizer v7.13 (Malvern Panalytical Ltd., Malvern, UK), and were performed at 25°C with a detection angle degree of 173.

### Electron Microscopy

Field emission scanning electron microscopy (FESEM) (Zeiss Gemini Ultra Plus, Oberkochen, Germany) was used to evaluate the particle size and morphology. ENCPs were diluted in a ratio between 1:100 and 1:1,000 in water, and then 1:1 with phosphotungstic acid (2% in water). A sample volume of 1 μL was placed on a copper grid with carbon films and, once dried, it was washed with 1 mL of ultrapure water. Dried samples were analyzed under the microscope using the InLens detector. Images with 50,000x magnification were taken for all the prototypes.

### Nanocomplex Stability in Cell Culture Media

The colloidal stability of the different ENCPs in cell culture media (RPMI + 10% FBS + 2% penicillin/streptomycin) was assessed at 37°C for up to 24 h. For this purpose, ENCPs were diluted 5 times in pre-warmed media, or water as the control, and particle size and PDI measured at 0, 4, and 24 h of incubation.

### Agarose Gel Retardation Assay

To qualitatively determine the amount of poly(I:C) within the ENCPs, samples were loaded in an agarose gel at 1% w/v in Tris Acetate-EDTA buffer (Sigma-Aldrich, MO, USA) before and after the incubation with an excess of heparin for poly(I:C) displacement. Each lane was loaded with 2.5 μg of poly(I:C) and with 1x SYBR®Gold nucleic acid stain (Invitrogen, CA, USA). For the displacement with heparin, 20:1 and 500:1 weight ratios of heparin (Sigma-Aldrich, MO, USA) to poly(I:C) were added for the C12r8 or pArg ENCPs, respectively, and incubated for 30 min at 37°C. Control lanes included a DNA 1 kb ladder (Invitrogen, CA, USA), and free poly(I:C) in the same conditions as the ENCPs. Gels were run for 30 min at 90 V in a Sub-Cell GT cell 96/192 (Bio-Rad Laboratories, CA, USA), evaluated with an UV transilluminator (Molecular Imager® Gel Doc™ XR, Bio-Rad Laboratories, CA, USA) and analyzed with Image Lab™ Software (Bio-Rad Laboratories, CA, USA).

For the release of poly(I:C), ENCPs were incubated in cell culture media, in a 1:1 (v/v) ratio, for 4 or 24 h, prior to been processed as described above. Free poly(I:C) exposed to the same conditions was used as the control.

### Human Primary Macrophage Differentiation

Human primary monocytes from blood of healthy donors were purified through density gradients, as previously reported (12, 41). M0 macrophages were obtained by culturing 1 × 10^6^ cells/mL human monocytes for 5 days in 5% FBS/RPMI supplemented with 25 ng/mL of recombinant human M-CSF (rhM-CSF; PeproTech, London, UK). M1 macrophages were polarized by stimulating M0 macrophages with LPS (100 ng/mL) (PeproTech, London, UK) and IFN-γ (50 ng/mL) (PeproTech, London, UK) for 24 h, and M2 macrophages were obtained by polarizing M0 macrophages with IL-4 (20 ng/mL) (PeproTech, London, UK) for 24 h. These cells were seeded in multiwell plates as indicated below for each experiment, and incubated at 37°C and 5% CO_2_. In all experiments, the final poly(I:C) dose employed was 5 μg/mL, with the exception of cell viability studies, where the specific doses are indicated.

### Cell Viability Studies

M0 and M2 human-derived macrophages were isolated and differentiated, and then seeded in 96-well plates at a density of 1 × 10^5^ cells/well. Cells were treated with poly(I:C), in solution or nanocomplexed, at poly(I:C) concentrations of 1, 5, 10, and 20 μg/mL. Macrophages were incubated with the nanosystems at indicated times, and cell viability was determined by Alamar Blue assay (Invitrogen, CA, USA), following manufacturer's protocol. Fluorescence intensity was measured in a plate reader (Synergy H4, BioTek, VT, USA), setting the λ_abs_ at 560 nm and the λ_em_ at 590 nm. Non-treated macrophages were used as controls and considered as 100% cell viability. Cell viability was calculated according to Equation (1).

(1) $$\begin{array}{l} \%\;Cell\;viability=\;\left(1-\frac{Fluorescence}{Control\;fluorescence}\right)\times 100 \end{array}$$

### Uptake Studies

Human monocytes were purified and polarized toward M0 macrophages as described in section Human Primary Macrophage Differentiation. These cells were seeded at a density of 1 × 10^6^ cells/well in low-attachment 24-well plates (Corning, ME, USA), and 0.5 mL of fresh RPMI media containing poly(I:C), either free or nanocomplexed, were added to them. The final poly(I:C) dose per well was of 5 μg/mL of poly(I:C), of which 0.25 μg/mL were poly(I:C)-rhodamine. After 24 h of incubation, cells were detached from the wells with trypsin-EDTA. Cells were then washed one time with FACS buffer (PBS 1% BSA) and fixed in FACS Fix (PBS 1% PFA) for 20 min at 4°C. Cell suspensions were centrifuged at 1,750 rpm for 10 min and 4°C. The supernatants were then discarded, and cells re-suspended in 300 μL of FACS buffer (PBS 1% BSA). Treated macrophages were analyzed by flow cytometry using a BD LSR Fortessa^TM^ (BD Biosciences, CA, USA), and the resulting data analyzed by FACS Diva software (BD Biosciences, CA, USA), determining the mean fluorescence intensity (MFI) of rhodamine-positive cells. Results were expressed as fold change in comparison to the free poly(I:C)-rhodamine.

### Co-localization Studies

Purified human monocytes were seeded in 24-well plates with a round glass coverslip at the bottom, at a density of 1 × 10^6^ cells/well in 1 mL of complete RPMI supplemented with M-CSF (25 ng/ml) to differentiate them to M0 macrophages. At day 5, 10 μL/well of CellLight® lysosome-GFP, BacMam 2.0 (Molecular Probes, OR, USA) were added. At day 6, coverslips were washed and poly(I:C), free or in pArg:pIC ENCPs, was added in 500 μL of fresh complete RPMI for 2 or 8 h. The final poly(I:C) dose per well was 5 μg/mL, of which 0.25 μg/mL were poly(I:C)-rhodamine. After incubation, cells were washed one time with PBS, nuclei were stained with DAPI and cells were fixed in 4% PFA (in PBS) for 10 min at room temperature. The glass coverslip was then recovered, mounted and analyzed with a Leica TCS SP8 3X SMD confocal microscope (Leica Microsystems, Wetzla, Germany). Signal co-localization was quantified with IMARIS software (Oxford Instruments, Abingdon, UK).

### Macrophage Surface Marker Expression

M0 or M2 human-derived macrophages were seeded in 24-well low-attachment plates at a density of 1 × 10^6^ cells/well and incubated in 5% FBS supplemented RPMI for 48 h at 37°C. Macrophages were then incubated with 5 μg/mL of poly(I:C), either in solution or nanocomplexed. M1 macrophages were also used as control, and M2 macrophages were polarized toward M1 phenotypes upon incubation with LPS/IFN-γ. Prior to their staining, cells were washed, collected and resuspended in FACS buffer (PBS 1% BSA). They were then stained with APC-mouse anti-human HLA-DR (552764, BD Biosciences, CA, USA), FITC-mouse anti-human mannose receptor CD206 (551135), anti-human CD163-BV421 (562643), CD80 APC-H7-mouse anti-Human CD80 (Clone L307.4; 561134) and anti-human CD68-PE (556078) (all from BD Biosciences, CA, USA). Cells were analyzed by flow cytometry on FACS Canto II Instrument (BD Biosciences, CA, USA) and the generated data by FACS Diva software. The gated cells were plotted on APC (CD80), PerCP (MHC II), Pacific Blue (CD163) or FITC (CD206) and analyzed for mean fluorescent intensity (MFI). Results were expressed as fold change in comparison to untreated M0 macrophages.

### Secretion of Chemokines

The levels of the chemokines CXCL10 and CCL5 were measured by commercially available ELISA kits following the manufacturer's instructions (R&D Systems, MN, USA). The supernatants were collected after 24 h of treatments.

### PCR

Total RNA was collected from macrophages with PureZOL™ RNA Isolation Reagent (Bio-Rad, CA, USA) and purified with Direct-zol RNA Miniprep kit (Zymo Research, CA, USA). From 1 μg total RNA, cDNA was synthesized by random priming with the High-Capacity cDNA Reverse Transcription kit (Applied Biosystems, CA, USA) according to the manufacturer's instructions. SYBR™ Green PCR Master Mix (Applied Biosystems, CA, USA) was used for Real-Time PCR on a QuantStudio 7 Flex Real Time PCR Systems (Applied Biosystems, CA, USA) according to the manufacturer instructions. The sequences of primer pairs were as follows: hGAPDH; 5′-AGA TCA TCA GCA ATG CCT CCT G-3′ and 5′-ATG GCA TGG ACT GTG GTC ATG-3′, hCCL5; 5′-TGC ATC TGC CTC CCC ATA TT-3′ and 5′- GAC CTT GCC ACT GGT GTA GAA A-3′, hIRF7; 5′- CCA CGC TAT ACC ATC TAC CTG G−3′ and 5′- GCT GCT ATC CAG GGA AGA CAC A−3′, hCD206; 5′- GGA GTG ATG GTT CTC CTG TTT-3′ and 5′- CCT TTC AGC TCA CCA CAG TAT T-3′. Cycling conditions: 10 min at 95°C, 40 cycles of 15 s at 95°C, and 1 min at 60°C. Data were normalized to *GAPDH* mRNA by subtraction of the cycle threshold (Ct) value of *GAPDH* mRNA from the Ct value of the gene (ΔCt). Fold difference was calculated by comparing the ΔCt with the ΔCt of untreated M0 macrophages (ΔΔCt).

### Cytotoxicity of Pre-treated Macrophages Toward PANC-1 Tumor Cell Line

The cytotoxicity of the pre-treated macrophages toward cancer cells was performed as described in a recent publication (12). Briefly, primary monocytes isolated from human healthy donors were stimulated with M-CSF in 5% FBS supplemented RMPI medium for 5 days. Then, macrophages were treated with the different ENCPs or free poly(I:C) in a dose of 5 μg/mL for 24 h. Alternatively, macrophages were treated with LPS/IFN-γ or IL-4 to polarize them toward M1 or M2 phenotypes. Macrophages were then washed and co-incubated for 2 days with 25,000 cells of a pancreatic cancer cell line (PANC-1), that were previously stained with Cell Trace Far Red (Invitrogen, CA, USA). The cells were trypsinized and fixed in FACS Fix for 20 min at 4°C for flow cytometry analysis using FACS Canto II Instrument (BD Biosciences, CA, USA). For the flow cytometry analysis, acquisition was set to 45 s and the number of high fluorescence intensity events (corresponding only to proliferating PANC-1 cells) were counted for each sample and normalized to the non-treated (M0) macrophages.

### Statistical Analysis

Data analysis was performed with GraphPad Prism version 7.0 (GraphPad Inc.). Statistical comparison was done using a two-way ANOVA followed by a Tukey's multiple comparison test; an ordinary one-way ANOVA followed by a Tukey's multiple comparison test; or a paired *t*-test when comparing only two sets of data. Data are expressed as the mean ± standard deviation (SD). *p-*values of 0.05 or less were considered statistically significant. In the *in vitro* experiments “*n*” represents the number of each macrophage population obtained from each blood donor. For the PCR results “*N*” represents the number of experimental replicates.

## Results and Discussion

The main objective of this study has been to develop a delivery carrier for poly(I:C) that would promote the polarization of macrophages toward an anti-tumoral M1-like phenotype. With this idea in mind, we selected different arginine-rich polymers and oligomers for the complexation of poly(I:C) and, then, we enveloped these positively charged nanocomplexes with pegylated polyglutamic acid (PEG–PGA) or hyaluronic acid (HA) to produce ENCPs. Following a rigorous characterization in terms of particle size, zeta potential and drug loading capacity, these ENCPs were evaluated *in vitro* for their biocompatibility, capacity to be internalized and ability to revert the polarization of human primary macrophages toward M1-like anti-tumoral phenotypes. Finally, the capacity of the macrophages treated with nanoformulated poly(I:C) to secrete T-cell attracting chemokines and to directly kill cancer cells was also assessed.

### Design and Development of Poly(I:C) Nanocomplexes

In the last decades, the formulation of anti-cancer drugs in nanosystems has been extensively studied with the aim of improving their accumulation in the tumor site, hence decreasing their off-target effects (42, 43). This research has led to a significant number of marketed nanoparticle-based anti-cancer drugs with an improved safety profile (42). In parallel, although at early stages, the development of nanosystems associating immunomodulators is already showing promising results at the preclinical level (44). At the same time, the formulation of polynucleotides within nanosystems is able to protect them from degradation (45). Bearing all this in mind, we have formulated poly(I:C) in the form of nanocomplexes enveloped with two biodegradable and stabilizing polymers (PEG–PGA and HA), known to facilitate the arrival to the tumor site (40, 46–53). Once in the tumor, a preferential uptake of the nanosystems by macrophages could be anticipated due to their high phagocytic capacity, as already described for both, targeted and non-targeted nanosystems (15, 54, 55). Considering our own previous results and also relevant literature in the field (46, 53), the targeted nanocomplexes should present particle sizes lower than 200 nm, with a pegylated or negative surface, and a high stability in relevant media.

#### Screening of Different Arginine-Rich Polymers

**Figure 1:**
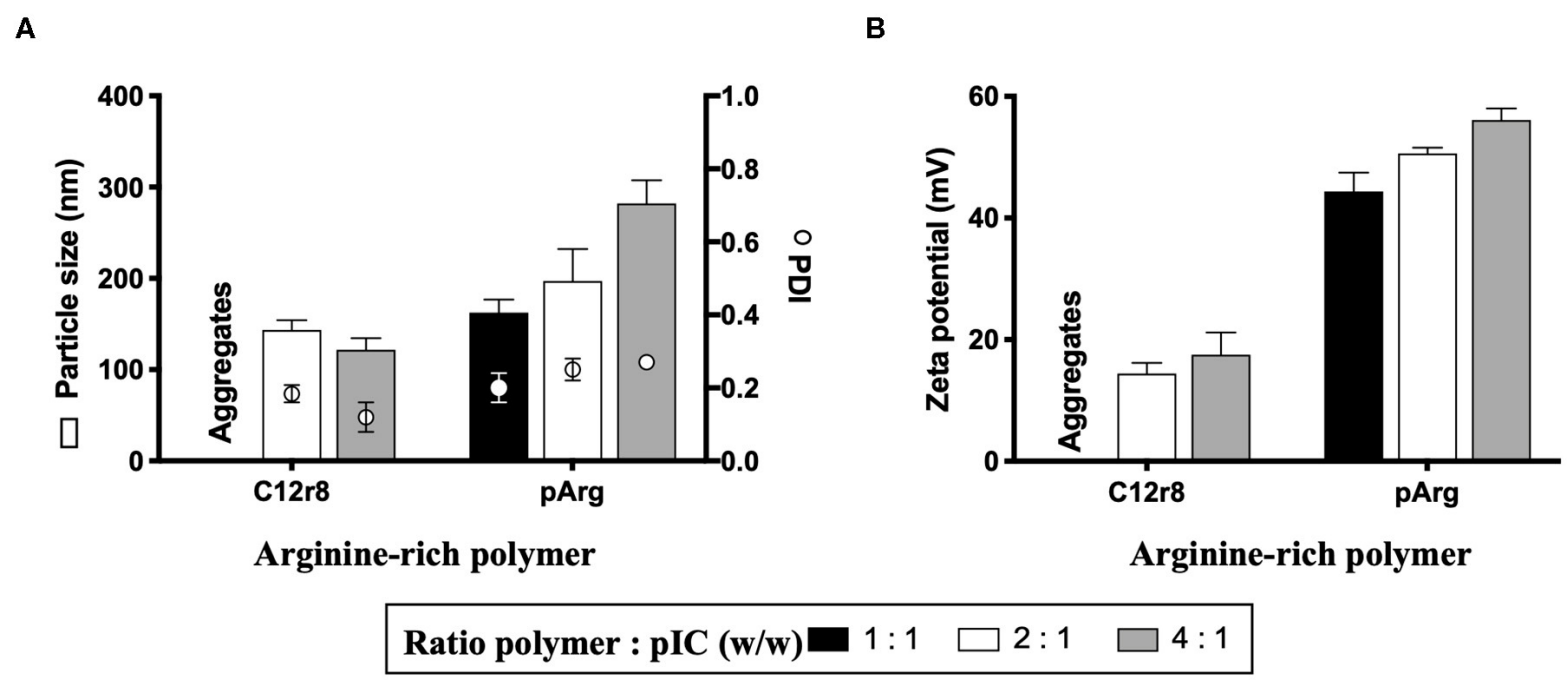
Screening of different arginine-rich polymers to form nanocomplexes with poly(I:C). Values of **(A)** particle size, PDI and **(B)** zeta potential of the nanocomplexes obtained for the different ratios tested. Values represent mean ± SD (*n* ≥ 3). C12r8, laurate-octaarginine; pArg, polyarginine; PDI, polydispersity index; pIC, poly(I:C); w/w, weight/weight.

As the first step in the development of a poly(I:C)-loaded nanoformulation and, based on a nanosystem recently reported by our group showing the capacity of modified octaarginine to complex polynucleotides (40), different positively-charged arginine-rich polymers were selected for poly(I:C) complexation. Oligo-arginines have been extensively employed for the delivery of different nucleic acids due to their CPP nature, which increases their uptake and, as a result, improves their therapeutic performance (37, 40, 56). For this purpose, and taking as a reference our previous work (40) and additional reports (57), two oligopeptides were selected: octaarginine (r8) and a hydrophobically-modified r8, that contains a laurate chain (C12r8). As a comparison, a higher MW arginine polymer, polyarginine (pArg), was also selected. Weight ratios arginine-rich polymer/oligomer to poly(I:C) ranging from 1:1 to 4:1 were evaluated in terms of their capacity to form nanocomplexes (Figure 1). In the case of the unmodified r8, despite previous works making use of this biomaterial, no stable nanosystems were obtained at the different ratios and thus, its use was discarded. On the contrary, the hydrophobized r8 could form stable nanocomplexes at ratios 2:1 and 4:1, a fact that indicates that the hydrophobic tail of C12r8 is critical for improving the stability of the resulting complexes (58, 59). In the case of the pArg-based nanocomplexes, all ratios yielded particles with sizes increasing from 150 to 300 nm, as the amount of pArg increased (Figure 1A). Positive surface charge values incremented following the same trend (Figure 1B).

In order to evaluate their suitability for *in vitro* testing, the nanocomplexes were incubated in cell culture media at 37°C, to determine their stability. In the case of C12r8, the particle size of the nanocomplexes increased upon incubation in cell culture media (Supplementary Figure 1A). On the contrary, pArg-based nanosystems (ratio 1:1) maintain their particle size under the same conditions (Supplementary Figure 1B). We hypothesized that the different stability of the nanocomplexes could be due to their different MWs, since the long positive chains of the pArg would offer a higher number of positive sites for binding the dsRNA, in comparison to the smaller chains of the C12r8 (60).

#### PEG–PGA-Enveloped Nanocomplexes

In order to improve the nanocomplexes stability, we applied the technology previously described by our group (40, 61), using different pegylated polyglutamic acid (PEG–PGA) copolymers for the envelopment of the nanocomplexes. The presence of PEG as the external layer of the system was intended to provide steric protection and increase its colloidal stability (62). Additionally, the combination of PEG and PGA as the outer layer of polymeric nanocapsules has already been shown to facilitate their access to the tumor site in a passive manner (46, 47), and to improve the stability of C12r8-based nanocomplexes in biologically relevant media (61, 63).

It has been extensively reported that both, the PEG layer density and its conformation, are two key aspects in determining the fate and stability of the nanosystems (48, 49). In this work, we investigated different parameters of the copolymers with the idea to optimize the enveloping process, namely (i) a branched or diblock conformation, (ii) the length of the PGA chain, and (iii) the PEG density. For this purpose, branched copolymers with two PGA lengths (10 and 30 units) and different PEG substitution degrees (10 and 30%) were studied. At the same time, two diblock copolymer conformations with PGA lengths of 10 and 30 units, and a PEG tail of 20 and 5 kDa MW, were also evaluated (Supplementary Figure 2).

For the enveloping process, we searched for the optimal amount of PEG–PGA for an efficient coating. For this, we evaluated two different amounts of PEG–PGA polymer over the C12r8:pIC. For a weight ratio C12r8:pIC:PEG–PGA 4:1:6, narrow particle sizes were obtained and, although surface charge was decreased, only the diblock PEG_5k_-PGA30 generated a zeta-potential inversion (Supplementary Figures 3A,B). Similarly, a lower amount of PEG—PGA (4:1:3) caused a slight particle size increase, and a moderate decrease in the surface charge, with no charge inversion for any of the conditions (Supplementary Figures 3A,B). It is interesting to mention that the branched PEG_5k_30–PGA10 did not produce an important change in the surface charge of any of the ENCPs (Supplementary Figure 3B), behavior already reported for similar systems (61). In this regard, we can speculate that the small size of the PGA chain, and the high number of PEG tails, might hinder the adequate interaction of the polymer with the systems, making this copolymer inadequate for an efficient coating of these nanocarriers.

To determine the efficiency of PEG–PGA envelopments in improving the ENCPs stability, the variation of their physicochemical properties in cell culture media was monitored (Supplementary Figures 4A,B). The results showed that both diblock copolymers were able to stabilize the nanocomplexes in a weight ratio C12r8:pIC:PEG–PGA 4:1:3 (Supplementary Figure 4B). Nevertheless, the ENCPs with the diblock PEG_5k_-PGA30 did not present as good short-term stability in storage conditions as the PEG_20k_-PGA10 (data not shown), which led us to discard the use of that copolymer. Similar C12r8 nanosystems enveloped with this PEG–PGA arrangement were also significantly stabilized (40, 63), which confirms that this diblock combination of a low number of PGA units (10) with a long PEG tail (20 kDa) provides good steric protection to a nanosystem.

Based on these results, the diblock PEG_20k_-PGA10 polymer was used for enveloping the pArg nanocomplexes, maintaining the pIC:PEG–PGA ratio, so that a more systematic comparison between the different nanocomplexes could be conducted. These ENCPs (weight ratio pArg:pIC:PEG–PGA 1:1:3) presented a particle size of 190 ± 15 nm, and a lower positive surface charge, when compared with the non-enveloped nanocomplexes (Supplementary Figures 3C,D). Furthermore, the colloidal stability in cell culture media showed that all ENCPs properties were maintained after 24 h of incubation (Supplementary Figure 4C), concluding that PEG_20k_-PGA10 has highly interesting properties for increasing the stability of nanosystems.

#### HA-Enveloped Nanocomplexes

Hyaluronic acid (HA) was also evaluated for the envelopment of the nanocomplexes, based on its anionic character and stabilization properties (50–52). Indeed, a recent report has claimed that HA coatings are able to decrease the adsorption of immunogenic proteins in comparison to other anionic coatings (64). Furthermore, HA-coated nanocapsules recently developed by our group showed an improved tumor accumulation after systemic administration (53). All these characteristics were expected to confer stability to the ENCPs, together with longer circulation times.

In line with this, several weight ratios of HA were evaluated to envelop the C12r8 and the pArg nanocomplexes. In the case of C12r8, the lowest amounts of HA led to aggregation, probably because the surface charges of the ENCPs were close to neutrality. For the other ratios, the ENCPs presented sizes of 150–200 nm and negative surface charges (Supplementary Figure 5).

When evaluating the stability in cell culture media, none of the C12r8 ENCPs were sufficiently stable after the envelopment with HA, reason why they were discontinued for the following experiments (Supplementary Figure 6A). Oppositely, for HA-enveloped pArg nanosystems, all ENCPs were stable after incubation in cell culture media (Supplementary Figure 6B). Among them, the weight ratio pArg:pIC:HA 1:1:1.5 showed the best properties in terms of its short-term stability (data not shown), and, therefore, was selected for further evaluation. Regarding the different stability of the HA-enveloped nanosystems, it is known that the presence of salts and the high ionic strength of the cell culture media can potentially disturb the ionic interactions governing the stability of some colloidal systems (65). Additionally, we hypothesized that the different MW of pArg and the C12r8 can cause a more tightly attachment of the HA coating in the case of the longer chains of the pArg, increasing their stability in cell culture media.

Overall, the conclusion from these envelopment tests is that the polymeric coating can significantly increase the stability of the nanocomplexes, but the process needs to be optimized in a case-by-case basis, being mainly determined by the nanocomplexes composition and the nature of the enveloping polymer.

### Association Capacity of Poly(I:C) to the Enveloped Nanocomplexes (ENCPs)

**Figure 2:**
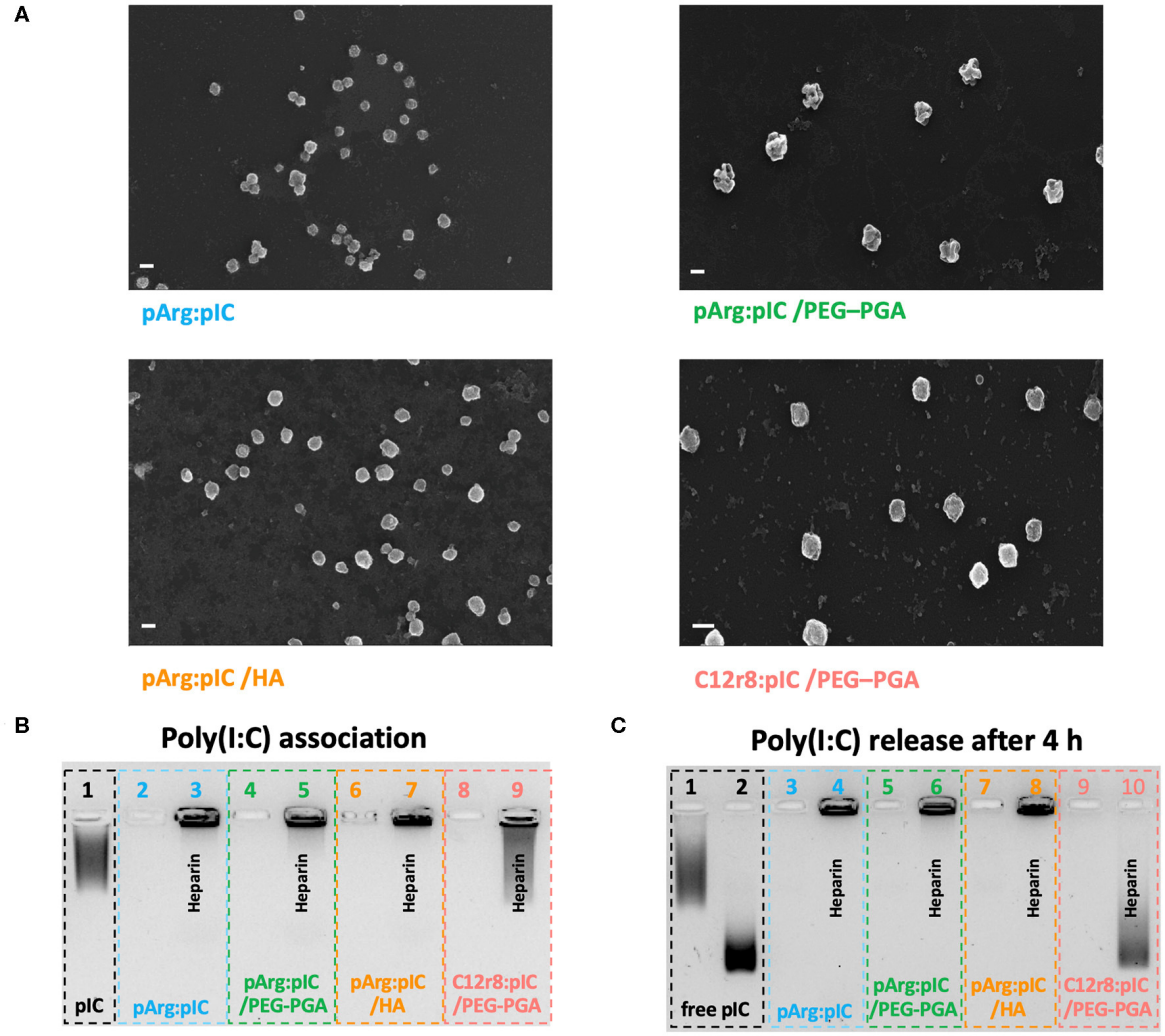
Physicochemical properties and poly(I:C) binding affinity of the selected nanocomplexes. **(A)** FESEM images of each of the four developed nanosystems. Values represent mean ± SD (*n* ≥ 12). Size bars represent 200 nm, and all images present a 50 K magnification. **(B)** Agarose gel retardation assay to evaluate the poly(I:C) binding capacity of the nanocomplexes. Lanes: (1) free poly(I:C), (2,4,6,8) are pArg:pIC, pArg:pIC/PEG–PGA, pArg:pIC/HA and C12r8:pIC/PEG–PGA nanocomplexes, respectively; and (3,5,7,9) are the corresponding nanocomplexes incubated with heparin. **(C)** Agarose gel retardation assay to evaluate the release and integrity of poly(I:C) after 4 h of incubation in cell culture media at 37°C. Lanes: (1) free poly(I:C) in solution and (2) in cell culture media; (3,5,7,9) are pArg:pIC, pArg:pIC/PEG–PGA, pArg:pIC/HA and C12r8:pIC/PEG–PGA nanocomplexes in cell culture media; and (4,6,8,10) are the same conditions incubated with heparin. C12r8, laurate-octaarginine; FESEM, field emission scanning electron microscopy; HA, hyaluronic acid; pArg, poly-arginine; PEG–PGA, pegylated polyglutamic acid; PDI, polydispersity index; pIC, poly(I:C); w/w, weight/weight.

After the screenings described in the precedent sections, a total of four ENCPs were selected to investigate their capacity to polarize macrophages: non-enveloped, diblock PEG_20k_-PGA10 enveloped and HA-enveloped pArg nanocomplexes (pArg:pIC ENCPs; pArg:pIC/PEG–PGA ENCPs and pArg:pIC/HA ENCPs, respectively) and diblock PEG_20k_-PGA10 enveloped C12r8 nanocomplexes (C12r8:pIC/PEG–PGA ENCPs). Their main physicochemical properties are summarized in Table 1. All ENCPs presented particle sizes between 150 and 200 nm, with low PDIs and surface charges ranging from highly positive (pArg:pIC), through neutral (C12r8:pIC/PEG–PGA) to negatively charged (pArg:pIC/HA). Remarkably, high loading values of poly(I:C) were obtained for the different systems (Table 1). Electron microscopy confirmed the size, homogeneity and spherical shape of the ENCPs (Figure 2A).

**Table 1:** Summary of the main physicochemical properties of the four enveloped nanocomplexes used in the *in vitro* experiments.

**Formulation**	**Ratio (w/w)**	**Particle size (nm)**	**PDI**	**ζ-Potential (mV)**	**Poly(I:C) loading (%)**
pArg:pIC	1:1	163 ± 14	0.20	+44 ± 3	50
pArg:pIC /PEG–PGA	1:1 :3	190 ± 14	0.18	+26 ± 8	20
pArg:pIC /HA	1:1 :1.5	157 ± 10	0.16	−15 ± 7	29
C12r8:pIC /PEG–PGA	4:1 :3	165 ± 14	0.07	+5 ± 1	12.5

*Mean ± SD, n ≥ 12. C12r8, laurate octa-arginine; HA, hyaluronic acid; pArg, polyarginine; PDI, polydispersity index; PEG–PGA, pegylated polyglutamic acid; pIC, poly(I:C); w/w: weight/weight*.

Secondly, the efficacy of the ENCPs to associate poly(I:C) was qualitatively evaluated. An agarose gel retardation assay confirmed that all nanosystems efficiently interacted with poly(I:C), with no free poly(I:C) detected, and the incubation with the competitor polyanion heparin was able to partially displace the cargo (Figure 2B). Moreover, the incubation of the nanocomplexed poly(I:C) in cell culture media during 4 or 24 h did not disrupt the interaction between poly(I:C) and pArg (Figure 2C and Supplementary Figure 7A, lanes 2–7). Instead, free poly(I:C) suffered a degradation when exposed to the cell culture media, as noted by the decrease in the MW (Figure 2C and Supplementary Figure 7A, lane 2). This degradation was probably caused by the RNases present in the media, since this degradation did not happen in water in the same conditions (Supplementary Figure 7B).

In the case of the C12r8:pIC/PEG–PGA ENCPs, poly(I:C) was not released upon incubation in cell culture medium, but after the displacement with heparin some degradation could be observed at 4 and 24 h (Figure 2C and Supplementary Figure 7A, lanes 9–10). We hypothesized that this degradation could be due to the interaction of the displaced poly(I:C) with the enzymes of the media (Supplementary Figure 7B), since the poly(I:C) inside the nanocomplexes is expected to be protected from enzymes. Therefore, these results demonstrate the capacity of the ENCPs to protect and prevent the premature release of poly(I:C).

### *In vitro* Toxicity of the Nanocomplexes

**Figure 3:**
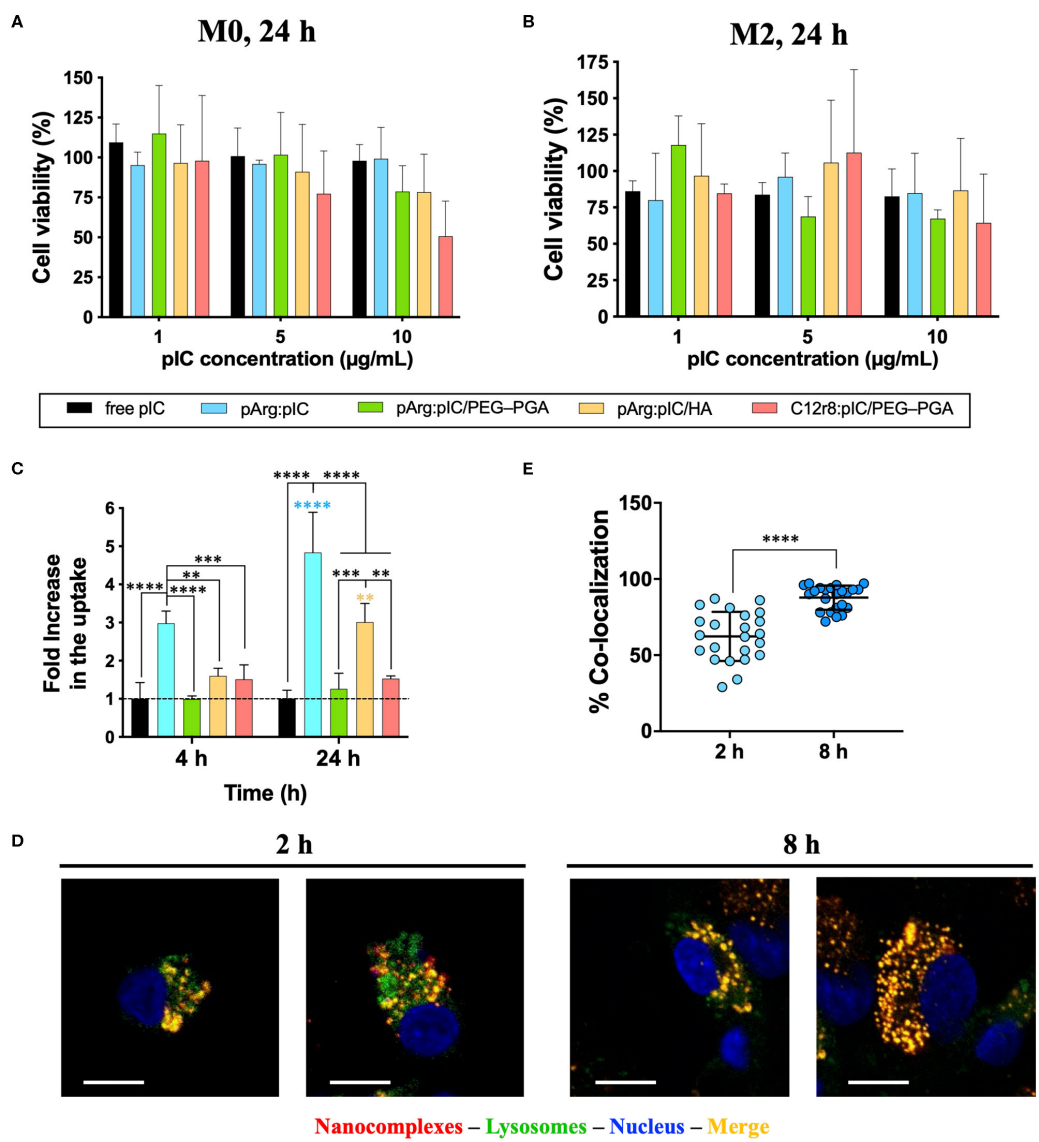
Toxicity, uptake and cellular localization of poly(I:C) and nanocomplexes. Toxicity toward **(A)** M0 and **(B)** M2 primary human monocyte-derived macrophages after 24 h of incubation. **(C)** FACS evaluation of rhodamine-labeled-poly(I:C) uptake by primary human monocyte-derived macrophages when included in the different nanocomplexes after 4 and 24 h of incubation, expressed as the fold increase in comparison to free poly(I:C), for a final poly(I:C) dose of 5 μg/mL. **(D)** Co-localization with the endosome of rhodamine-labeled pArg:pIC nanocomplexes after 2 and 8 h of incubation (100x magnification, size bars of 10 μm) evaluated by confocal microscopy. **(E)** Quantification of the co-localization of rhodamine-labeled pArg:pIC nanocomplexes with the endosome after 2 and 8 h of incubation, with a poly(I:C) dose of 5 μg/mL. Values represent mean ± SD (*n* ≥ 3). Statistical comparison was done using a two-way ANOVA followed by a Tukey's multiple comparison test. Statistically significant differences are represented as **p < 0.01, ***p < 0.005, and ****p < 0.001. C12r8, laurate-octaarginine; HA, hyaluronic acid; pArg, poly-arginine; PEG–PGA, pegylated polyglutamic acid; pIC, poly(I:C).

Considering that the target cells of the developed ENCPs are immune cells of the myeloid lineage, primary human monocyte-derived macrophages were used to evaluate the *in vitro* biocompatibility of the nanosystems. M0 or M2 macrophages were incubated with the selected ENCPs for different times. When M0 macrophages were exposed to different concentrations of free or nanocomplexed poly(I:C) for 24 h, minor toxicity values were observed for the lowest doses (1 and 5 μg/mL), with no significant differences among ENCPs (Figure 3A). At higher doses (10 μg/mL), the poly(I:C) nanocomplexed with C12r8 showed higher toxicity than the free dsRNA (Figure 3A). This increased toxicity could be caused by the higher amount of the polypeptide C12r8 in comparison to pArg for the same dose of poly(I:C) (weight ratio 4:1 and 1:1, respectively), and due to the intrinsic toxicity of CPPs (66). A similar tendency was observed for M2 macrophages, showing similar toxicities for all the ENCPs at 10 μg/mL of poly(I:C), while the lower doses were much better tolerated (Figure 3B). As expected, shorter incubation times produced negligible toxicities (Supplementary Figures 8A,C), while longer incubation times decreased cell viability (Supplementary Figures 8B,D).

Overall, a similar toxicity of ENCPs vs. the free poly(I:C) at 5 μg/mL toward macrophages cultured *in vitro* was observed. Only a higher toxicity was found for some ENCPs at higher concentrations and longer time points (48 h), which could be expected, due to a higher uptake of the nanocomplexes vs. the free drug, as described in the next results section (Figures 3C–E). Thus, a non-toxic dose of 5 μg/mL of poly(I:C) was selected for the following experiments, finding a compromise between biocompatibility and an effective dose.

### Uptake and Cellular Internalization of the Nanocomplexes by Macrophages

In order to bind to its intracellular receptor (TLR3), poly(I:C) must be internalized by the macrophages. Thus, we evaluated if the uptake of poly(I:C) was improved when included into the ENCPs. Using rhodamine-labeled poly(I:C), the ability of macrophages to internalize free and nanocomplexed poly(I:C), together with its localization inside the cells, were studied (Figures 3C–E). When complexed only with pArg, the uptake of poly(I:C) was highly improved at 4 h, and even more at 24 h. This effect could be related to the high positive surface charge of the ENCPs vs. free poly(I:C) (Figure 3C). Similarly, a higher uptake was also observed for the HA-ENCPs, probably associated to the affinity of HA to the CD44 receptor on the surface of macrophages (67). In the case of the two PEG–PGA ENCPs, the uptake was only slightly better than the free dsRNA (Figure 3C). These results could be caused by the effect of the PEG chains, which might decrease the interaction of the ENCPs with the cell membrane, thus reducing their uptake by macrophages, as reported before for other nanoparticles (63, 68).

The ultimate target of the developed poly(I:C) ENCPs is the intracellular endosomal receptor TLR3. In order to confirm that the nanocomplexed poly(I:C) was able to reach this receptor, we studied the localization of the free and nanocomplexed poly(I:C) once inside the cells, as a proof-of-concept. For this, pArg:pIC ENCPs containing rhodamine-labeled poly(I:C), and CellLight® were used to track the cargo and the endosomes, respectively. Confocal experiments demonstrated the presence of poly(I:C) (in red) inside the endosome (in green) after 2 and 8 h of incubation, confirming the co-localization of the drug and its target (Figures 3D,E).

With this set of experiments, we can conclude that the inclusion of poly(I:C) into ENCPs improves its uptake and internalization, allowing the drug to efficiently reach its endosomal target inside macrophages. The functional studies described below were taken as additional validation of the adequate interaction of poly(I:C) with its target receptor.

### Macrophage Polarization Toward a Pro-inflammatory Phenotype

**Figure 4:**
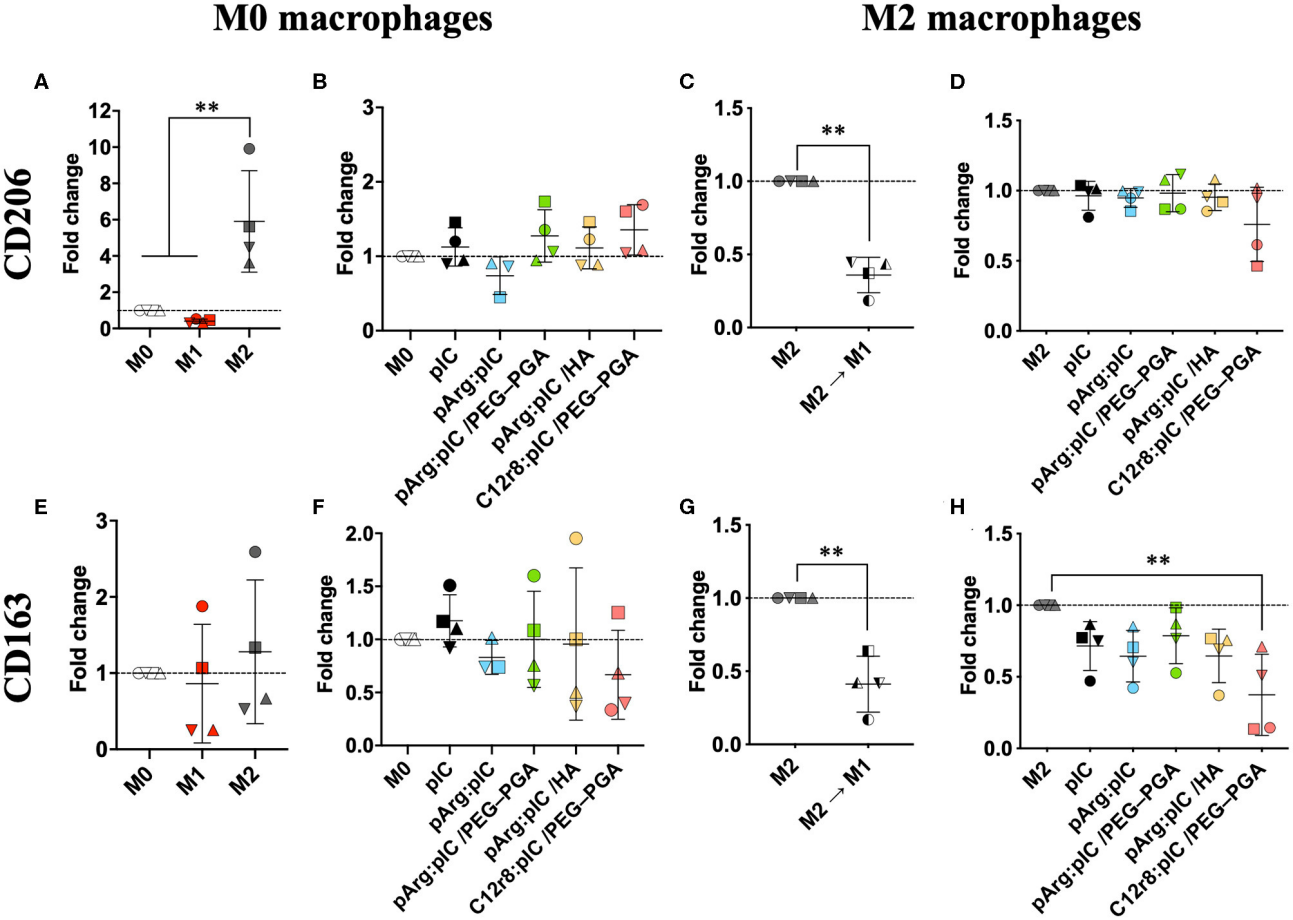
Polarization of M0 and M2 macrophages after treatment with free and nanocomplexed poly(I:C). Expression of the M2 markers **(A–D)** CD206 and **(E–H)** CD163 in ENCP-treated M0 and M2 macrophages, in comparison to the prototypic phenotypes evaluated by FACS. M2 M1 represents M2 macrophages that were treated with LPS + IFN-γ for their M1 polarization. Macrophages were incubated with the treatments for 48 h, and the poly(I:C) dose used was 5 μg/mL. Each symbol shape represents a different donor. Values are shown as mean ± SD (*n* ≥ 3). Statistical comparison was done using an ordinary one-way ANOVA followed by a Tukey's comparison test between groups; or a paired *t*-test for **(C,G)**. Statistically significant differences are represented as **p < 0.01. C12r8, laurate-octaarginine; HA, hyaluronic acid; pArg, poly-arginine; PEG–PGA, pegylated polyglutamic acid; pIC, poly(I:C).

The interaction of poly(I:C) with the endosomal TLR3 triggers an immune response through the TRIF pathway, and the subsequent activation of type I IFN genes (69). This should lead to a decreased expression of the M2-like features (e.g., CD206 and CD163); while M1 pro-inflammatory markers such as CD80 and MHCII should be increased (Figure 4 and Supplementary Figure 9). Therefore, we analyzed the presence of both M1 and M2 markers on the surface of macrophages 48 h after exposure to nanocomplexed poly(I:C) (5 μg/mL) (9, 11). As controls, M0 or M2 macrophages were polarized toward the M1 prototypic phenotype by treatment for 48 h with LPS and IFN-γ; or with IL-4 for inducing the prototypic M2 phenotype. No significant changes were detected in the mannose receptor CD206 upon treatment with free poly(I:C) or the ENCPs, in comparison to the untreated cells (Figures 4B,D); while it was overexpressed in the case of prototypic M2 macrophages (Figures 4A,C). Nevertheless, PCR analysis showed that the *CD206* mRNA levels for M0 macrophages exposed for 8 h to any of the ENCPs were indeed decreased (Supplementary Figures 10A,B). The difference observed between flow cytometry and PCR analysis might be due to the fact that protein receptors stay for long times in the membrane, therefore, the presence of CD206 receptor on the surface of macrophages could not be representative of the gene downregulation induced by the drug at the times of analysis. Meanwhile, this change could be already be seen in the *CD206* mRNA levels, as already reported for similar cases (12).

In the case of the scavenger receptor CD163, its presence on the surface of macrophages was lower upon treatment with any of the ENCPs, with significant differences in M2 macrophages treated with C12r8:pIC/PEG–PGA ENCPs (Figures 4E–H). This slight decrease of CD163 in macrophages exposed to ENCPs could be related to the polarization of macrophages toward the M1 phenotype, although we cannot discard that it might also be related to an involvement of this scavenger receptor in the uptake of the ENCPs. Further experiments would be required to fully understand the interaction of CD163 with the ENCPs.

**Figure 5:**
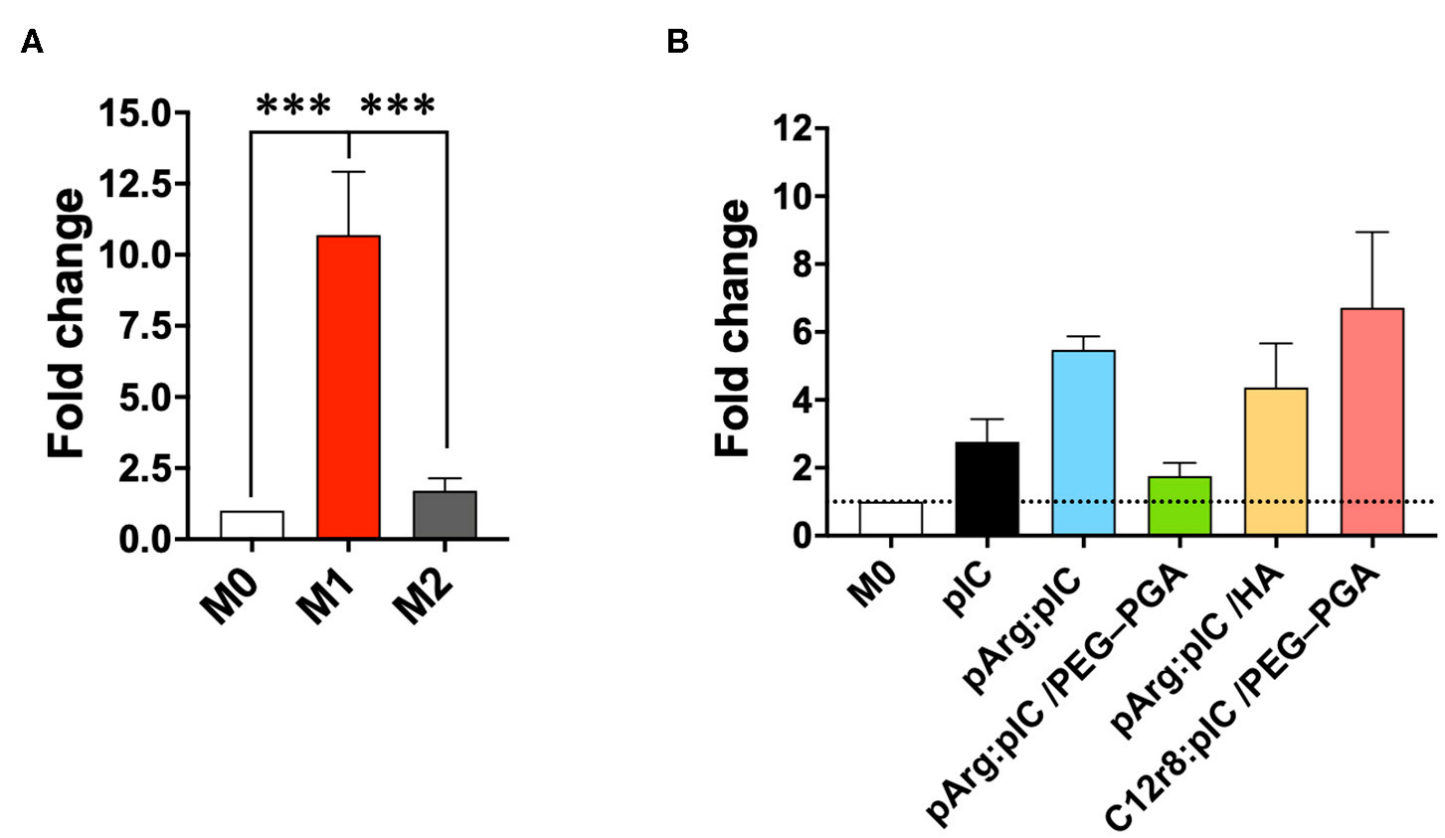
mRNA production of different M1/M2 associated factors. Fold change in the mRNA levels of *IRF7* in **(A)** prototypic M1/M2 macrophages and in **(B)** M0 macrophages treated with the different nanocomplexes after 8 h of incubation. The dose of poly(I:C) was 5 μg/mL. Values represent mean ± SD (*N* = 4). Statistical comparison was done using an ordinary one-way ANOVA followed by a Tukey's comparison test between groups. Statistically significant differences are represented as ***p < 0.005. C12r8, laurate-octaarginine; HA, hyaluronic acid; pArg, poly-arginine; PEG–PGA, pegylated polyglutamic acid; pIC, poly(I:C).

Overall, no significant changes in CD80 and MHCII were observed in the surface of M0 or M2 macrophages upon treatment with free or nanocomplexed poly(I:C) (Supplementary Figures 9B,D,F,H). Only a slight increase for the M1 markers CD80 and MHCII, was observed in macrophages exposed to C12r8:pIC/PEG–PGA ENCPs, but with no significant differences (Supplementary Figures 9B,D,F,H). A similar tendency was observed in the M1 controls (Supplementary Figures 9A,C,E,G). Furthermore, we also analyzed the levels of *IRF7* mRNA by PCR, a key molecule in the TRIF signaling pathway, which is triggered downstream of TLR3 activation (70). These experiments showed a higher level of *IRF7* mRNA in macrophages treated either with free or nanocomplexed poly(I:C) vs. M0 and M2 prototypic macrophages (Figure 5). In fact, these results correlated with the ones of the ENCPs uptake (Figure 3C).

As a whole, these results show a limited ability of free or nanocomplexed poly(I:C) to modulate the ratio of M1/M2 receptors on the surface of prototypical M0 or M2 macrophages, confirming the results recently published regarding the polarization capacity of poly(I:C) and imiquimod *in vitro* (12). The dynamic turnover of all these receptors probably hampers their precise quantification to assess the M1/M2 phenotypes *in vitro*. On the basis of these data, and being conscious that the ability of poly(I:C) to polarize macrophages toward M1-like anti-tumoral phenotypes can be better evaluated by conducting functional assays, we decided to test the ability of macrophages to secrete chemokines involved in the recruitment of T cells and the cytotoxic potential of pre-treated macrophages toward cancer cells.

### Improved T Cell Recruitment Capacity

**Figure 6:**
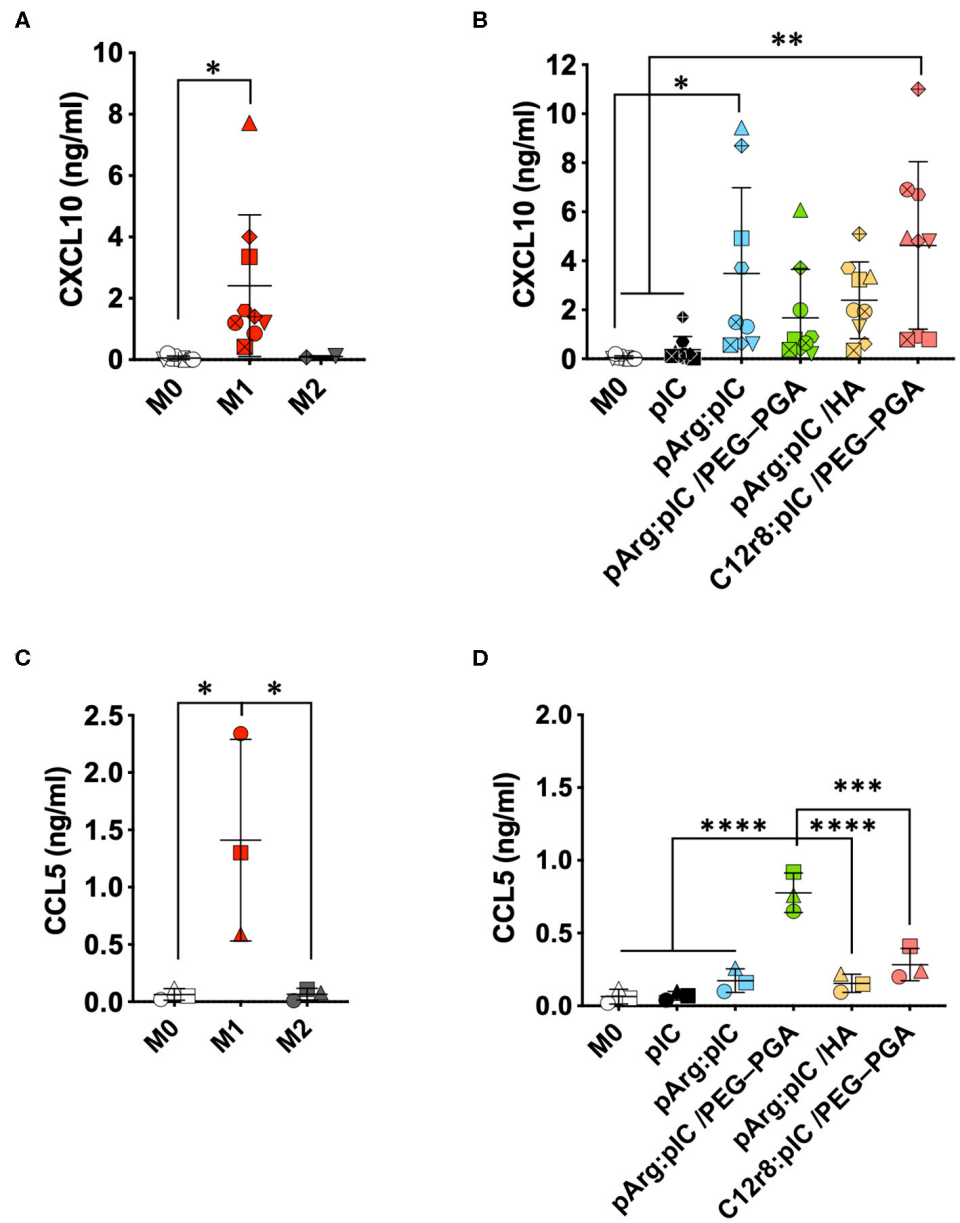
Secretion of the T cell attracting chemokines upon treatment with the poly(I:C) nanocomplexes. **(A,B)** CXCL10 secretion in **(A)** prototypic macrophages and in **(B)** M0 macrophages treated with the different nanocomplexes after 24 h of incubation. **(C,D)** CCL5 secretion in **(C)** prototypic macrophages and in **(D)** M0 macrophages treated with the different nanocomplexes after 24 h of incubation. Poly(I:C) was used at the final dose of 5 μg/mL. Each symbol shape represents a different donor. Values represent mean ± SD (*n* ≥ 3). Statistical comparison was done using an ordinary one-way ANOVA followed by a Tukey's comparison test between groups. Statistically significant differences are represented as *p < 0.05, **p < 0.01, ***p < 0.005, and ****p < 0.001. C12r8, laurate-octaarginine; HA, hyaluronic acid; pArg, poly-arginine; PEG–PGA, pegylated polyglutamic acid; pIC, poly(I:C).

Cytotoxic T cells (CTLs) are also important players in the anti-tumoral immune response (71, 72). CXCL10 and CCL5 are two key chemokines implicated in the recruitment of these CTLs by macrophages in order to fight against the cancer cells. Thus, we have evaluated the secretion of these chemokines by macrophages exposed to the ENCPs. We found a higher production of CXCL10 and CCL5 by macrophages treated with ENCPs vs. free poly(I:C) and the control (non-treated M0 macrophages) (Figure 6 and Supplementary Figure 11). Importantly, the levels of CXCL10 stimulated by the ENCPs were similar to the ones observed for M1 macrophages at 24 h, or even higher, in the case of the pArg:pIC and C12r8:pIC/PEG–PGA ENCPs (Figures 6A,B). Regarding CCL5, a minor increase in the secretion of this chemokine was observed after treatment with the ENCPs, which was only significantly higher for the pArg:pIC/PEG–PGA ENCPs (Figures 6C,D). In addition, *CCL5* mRNA levels further confirmed the stimulation of CCL5 production upon treatment with ENCPs, vs. the free poly(I:C) (Supplementary Figures 10C,D).

Altogether, these results indicate that, even though nanocomplexed poly(I:C) does not provoke an important change in the surface marker expression of macrophages, other anti-tumoral features such as the secretion of T cell-recruiting chemokines, was greatly improved.

### Increased Ability of Pre-treated Macrophages to Kill Tumor Cells

**Figure 7:**
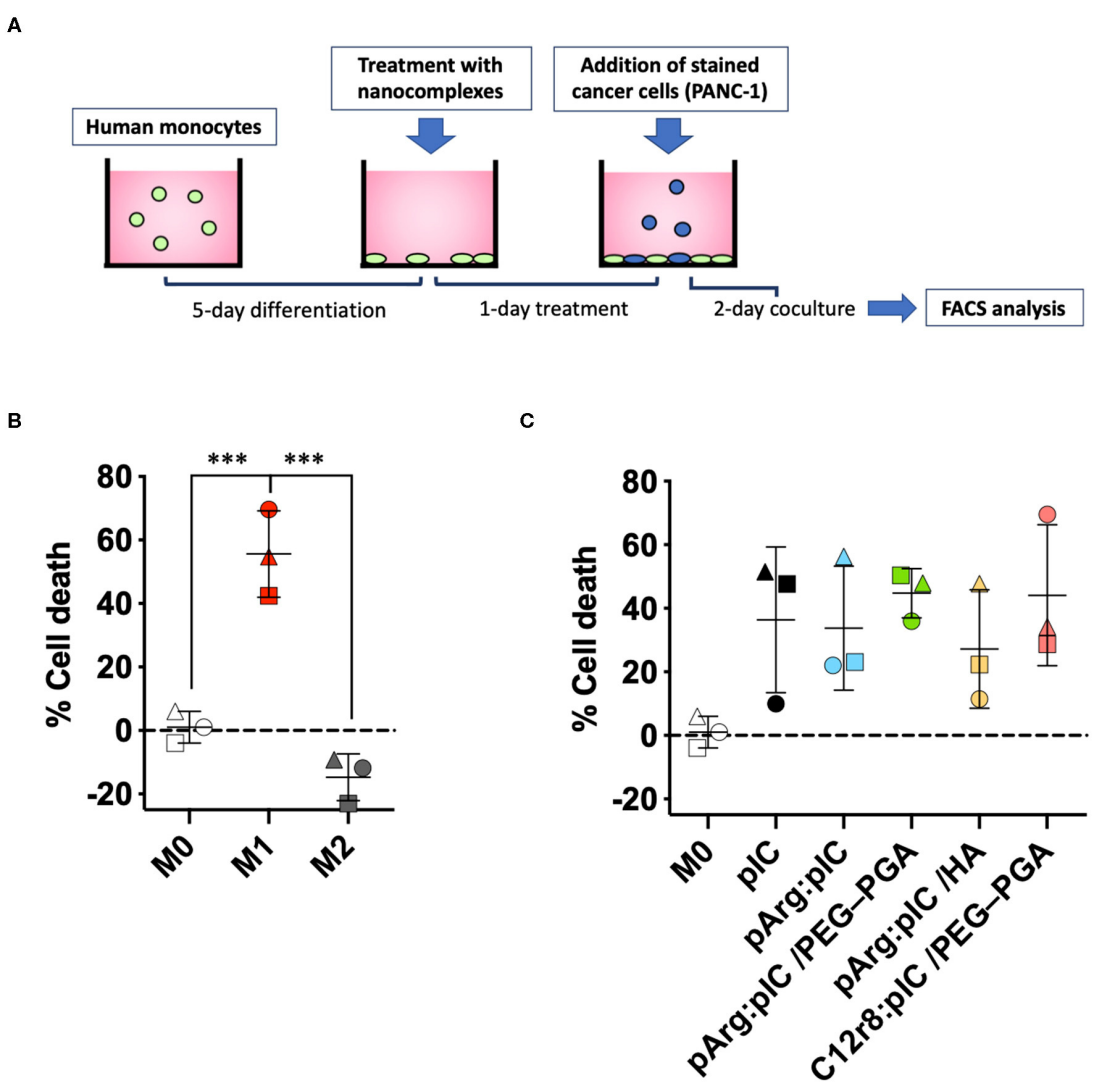
Macrophage cytotoxicity toward PANC-1 cancer cells after pre-treatment with the different nanocomplexes. **(A)** Schematic representation of the *in vitro* model for the determination of the killing capacity of pre-treated macrophages. **(B,C)** % of cancer cell death caused by **(B)** the prototypic macrophages or **(C)** M0 macrophages pre-treated with free or nanocomplexed poly(I:C). Poly(I:C) was used at the final dose of 5 μg/mL. Each symbol shape represents a different donor. Values represent mean ± SD (*n* ≥ 3). Statistical comparison was done using an ordinary one-way ANOVA followed by a Tukey's multiple comparison test between groups. Statistically significant differences are represented as ***p < 0.005. C12r8, laurate-octaarginine; HA, hyaluronic acid; pArg, poly-arginine; PANC-1, pancreatic cancer cells; PEG–PGA, pegylated polyglutamic acid; pIC, poly(I:C).

Besides their role in activating the immune system to fight cancer, anti-tumoral macrophages have also the capacity to directly kill tumor cells (73). To assess the potential of the poly(I:C) ENCPs to polarize macrophages toward M1-like anti-tumoral phenotypes, we performed a functional assay to evaluate their ability to kill tumor cells (Figure 7A). For this, M0 macrophages were treated with the different ENCPs during 24 h, or were differentiated to prototypical M1 or M2 phenotypes used as controls. These pre-treated macrophages were then co-cultured with stained pancreatic cancer cells (PANC-1) for 48 h. As expected, PANC-1 cells proliferated 15% more in co-culture with M2 macrophages, compared to non-polarized M0 macrophages (Figure 7B). On the other side, as a positive control, M1 macrophages presented a 60% increased ability to kill the cancer cells, when compared to M0 macrophages (Figure 7B). In the case of macrophages pre-treated with free and nanocomplexed poly(I:C), a 30–40% increase in their cytotoxicity toward cancer cells was observed vs. the untreated macrophages (Figure 7C). Considering that nanocomplexed poly(I:C) performed as well as the free drug, we can confirm that the dsRNA inside the nanocomplexes remained active.

## Conclusions

**Figure 8:**
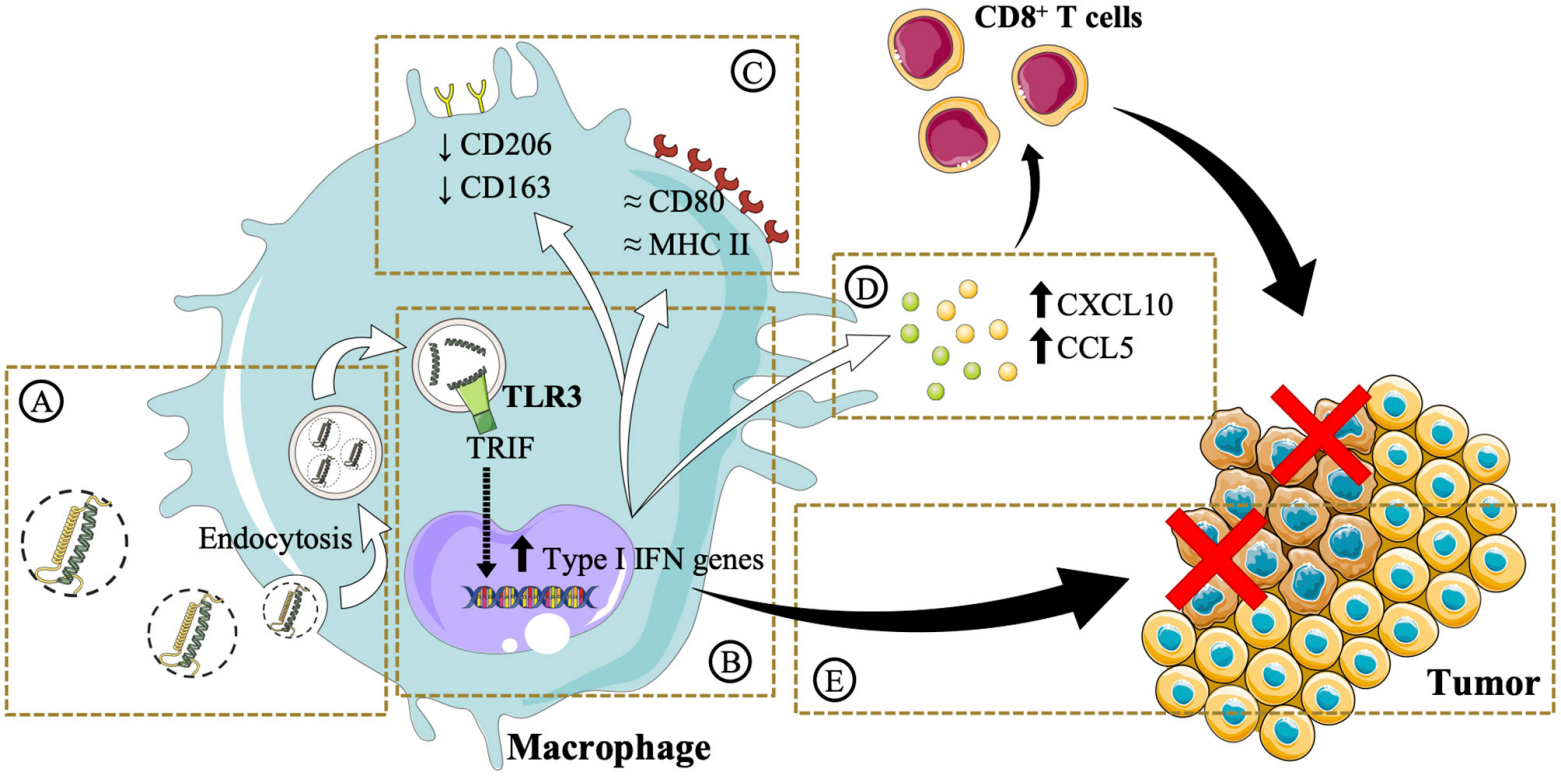
Schematic illustration of the *in vitro* effects of the poly(I:C) ENCPs developed in this study. **(A)** Upon interaction with macrophages, poly(I:C) nanocomplexes are taken up. **(B)** This process allows poly(I:C) to reach its target receptor TLR3, found in the endosomes. It is expected that this interaction activates the TLR3 and the TRIF pathway, stimulating the upregulation of type I IFN genes. **(C)** The expression of M2 (CD206 and CD163) surface markers was slightly decreased, while M1 (CD80 and MHC II) markers are not substantially modified. Nevertheless, **(D)** CXCL10 and CCL5 chemokines, involved in the attraction of CD8 T cells to the tumor microenvironment, are secreted. **(E)** The direct cytotoxicity of macrophages toward cancer cells is also enhanced. Images were reproduced from Servier Medical Art under a Creative Commons Attribution 3.0 Unported License https://creativecommons.org/licenses/by/3.0.

This work highlights the importance of a rational design in the development of poly(I:C) nanocomplexes to maintain the efficacy of the free drug while increasing its stability for *in vivo* administration. The complexation of poly(I:C) with arginine-rich polymers and their subsequent envelopment with either PEG–PGA or HA resulted in the formation of ENCPs with adequate physicochemical and stability properties. This delivery strategy facilitated the accumulation of poly(I:C) in the endosomal compartments, where the TLR3 is localized (Figures 8A,B). Minor changes in surface marker expression were detected, probably due to the dynamic turnover of these surface receptors (Figure 8C). However, in agreement with an improved poly(I:C) delivery, macrophages pre-treated with nanocomplexed poly(I:C) presented an enhanced secretion of T-cell attracting chemokines, which are critical for triggering effective anti-tumoral immune responses (Figure 8D). Moreover, macrophages pre-treated with either free or nanocomplexed poly(I:C) presented an improved capacity to directly kill cancer cells (Figure 8E). Altogether, these results provide evidence of arginine-based poly(I:C) nanocomplexes as a potential strategy for the M1-polarization of macrophages, that could be of advantage in the setting of cancer immunotherapy. Further *in vivo* biodistribution and anti-tumoral efficacy studies will help to elucidate whether these *in vitro* results are translatable, and if the systemic toxicity of the free dsRNA is indeed decreased.

## Data Availability Statement

The datasets generated for this study are available on request to the corresponding author.

## Author Contributions

TD, CA, and FT have contributed to the design, acquisition, analysis, interpretation of data, and the drafting and the revision of the work. FM has contributed to the design, acquisition, analysis, and interpretation of data. PA, MA, and JC-C have contributed to the design, the drafting, and the revision of the work. All authors contributed to the article and approved the submitted version.

## Conflict of Interest

The authors declare that the research was conducted in the absence of any commercial or financial relationships that could be construed as a potential conflict of interest.

## Abbreviations

C12r8: laurate octaarginine
CPP: cell penetrating peptide
ENCP: enveloped nanocomplex
HA: hyaluronic acid
pArg: polyarginine
PEG–PGA: pegylated polyglutamic acid
TAM: Tumor-associated macrophage.
