# Supplementary material for: Arginine-Based Poly(I:C)-Loaded Nanocomplexes for the Polarization of Macrophages Toward M1-Antitumoral Effectors
Source: Front Immunol. 2020 Jul 7;11:1412. doi: 10.3389/fimmu.2020.01412 (PMC7358452; doi:10.3389/fimmu.2020.01412)
Supplement: Supplementary file 1 [file Data_Sheet_1.PDF]

frontiers

Supplementary Material

Supplementary Table 1. Summary of the weight ratio arginine-rich polymer to poly(I:C) tested, and the final concentration of each component.

(+) polymer	Ratio (+) polymer: poly(I:C) (w/w)	(+) polymer (mg/mL)	Poly(I:C) (mg/mL)
r8; C12r8; pArg	1:1	0.33	0.33
	2:1	0.33 / 0.67	0.17 / 0.33
	4:1	0.67 / 1.33	0.17 / 0.33

C12r8, laurate-octaarginine; pArg, poly-arginine; r8, octaarginine; w/w, weight/weight.

Supplementary Table 2. Final concentrations of poly(I:C), arginine-rich polymers and polyglutamic pegylated acid in the enveloped nanocomplexes (ENCPs)

(+) polymer	Ratio (+) polymer: poly(I:C) : PEG–PGA (w/w)	(+) polymer (mg/mL)	Poly(I:C) (mg/mL)	PEG–PGA (mg/mL)
C12r8	4:1:6	0.67	0.17	1.00
	4:1:3	1.33	0.33	1.00
pArg	1:1:3	0.33	0.33	1.00

C12r8, laurate-octaarginine; pArg, poly-arginine; PEG–PGA, pegylated polyglutamic acid; w/w, weight/weight.

Supplementary Table 3. Final concentrations of poly(I:C), complexing polymers and hyaluronic acid in the enveloped nanocomplexes (ENCPs)

(+) polymer	Ratio (+) polymer: poly(I:C): HA (w/w)	(+) polymer (mg/mL)	Poly(I:C) (mg/mL)	HA (mg/mL)
C12r8	4:1:1.5	0.67	0.17	0.25
	4:1:3	0.67	0.17	0.50
	4:1:6	0.67	0.17	1.00
pArg	1:1:1.5	0.17	0.17	0.25
	1:1:3	0.17	0.17	0.50
	1:1:6	0.17	0.17	1.00

C12r8, laurate-octaarginine; HA, hyaluronic acid; pArg, poly-arginine; w/w, weight/weight.

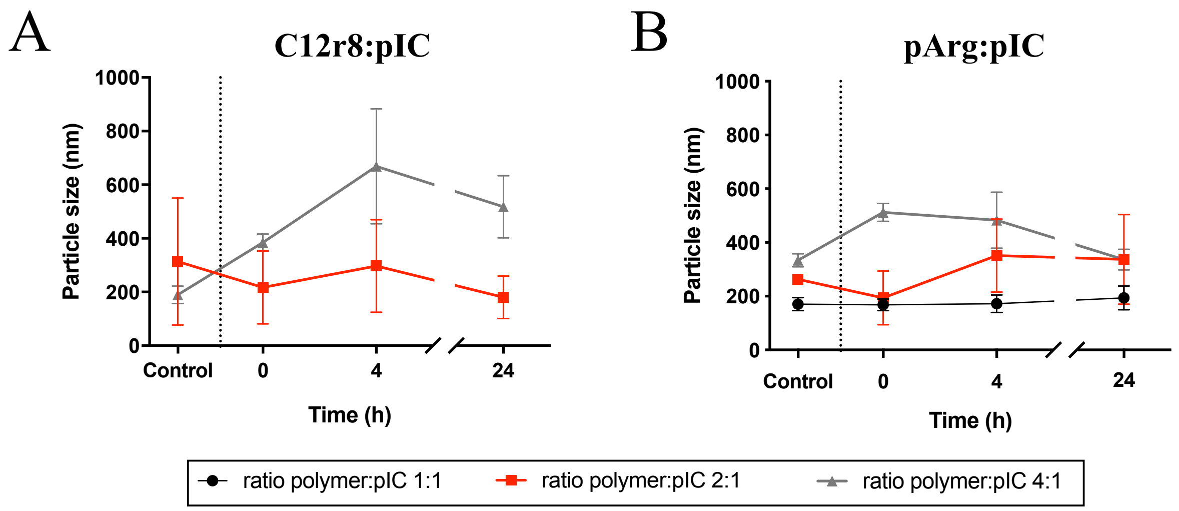

Supplementary Figure 1. Stability of the non-enveloped nanocomplexes in cell culture media. Evolution of particle size of **(A)** C12r8-based nanocomplexes and **(B)** pArg-based nanocomplexes up to 24 h of incubation in cell culture media at 37 °C, in comparison with the nanocomplexes in water (control). Values represent mean ± SD (n ≥ 3). C12r8, laurate-octaarginine; pArg, poly-arginine; pIC, poly(I:C).

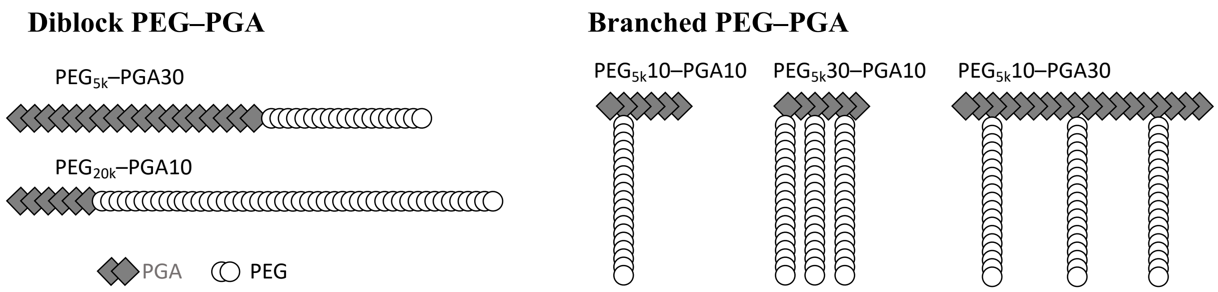

Supplementary Figure 2. Schematic representation of the PEG–PGA structures tested in these studies.

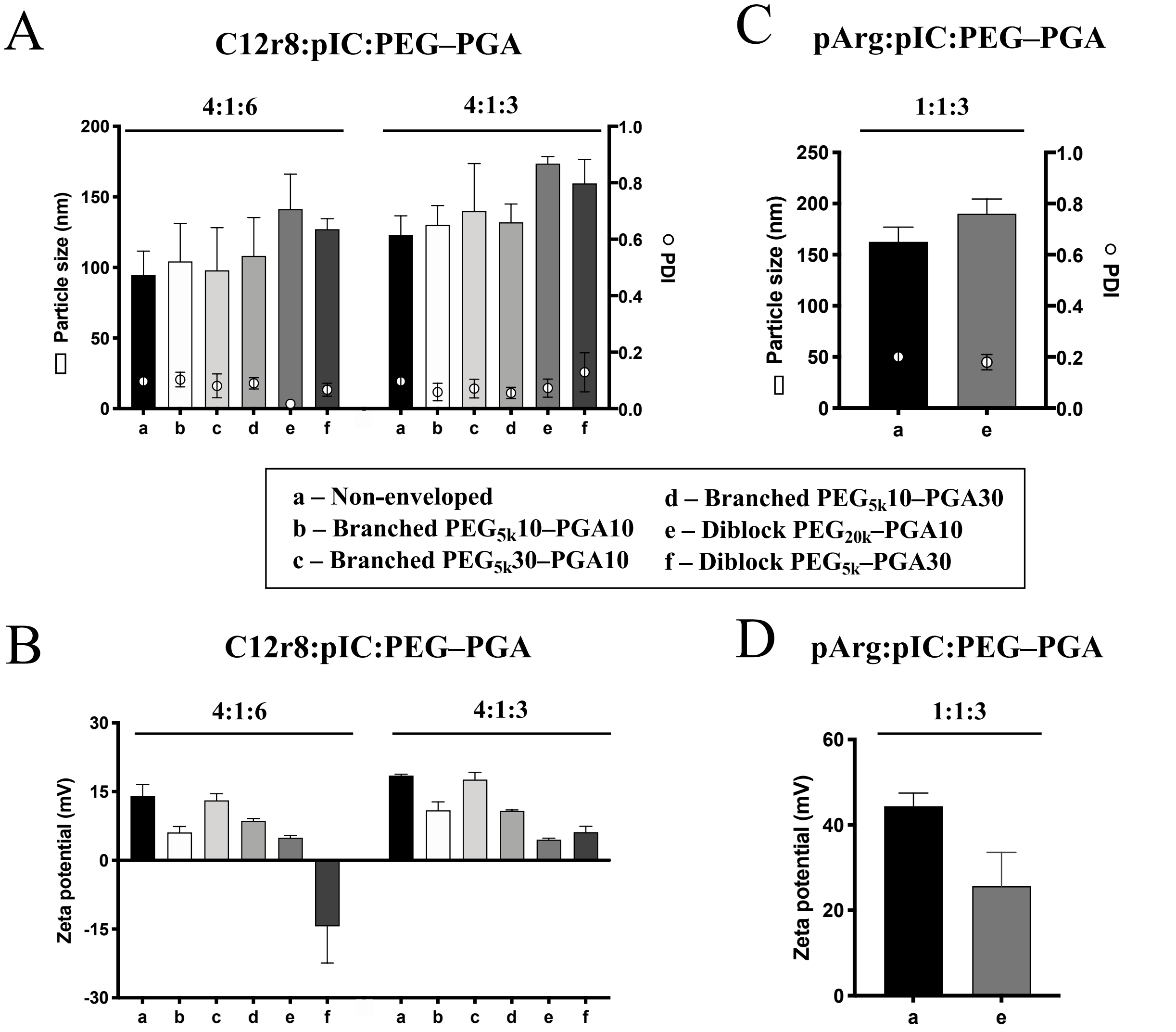

Supplementary Figure 3. Envelopment of poly(I:C)-loaded nanocomplexes with PEG–PGA. **(A)** Particle size, PDI and **(B)** zeta potential of the C12r8 nanocomplexes yielded from the different ratios and types of PEG–PGA tested. **(C)** Particle size, PDI and **(D)** zeta potential of the pArg nanocomplexes resulted of the envelopment with the most successful PEG–PGA copolymer selected from A,B. Values represent mean ± SD (n ≥ 3). C12r8, laurate-octaarginine; PEG–PGA, pegylated polyglutamic acid; PDI, polydispersity index; pIC, poly(I:C).

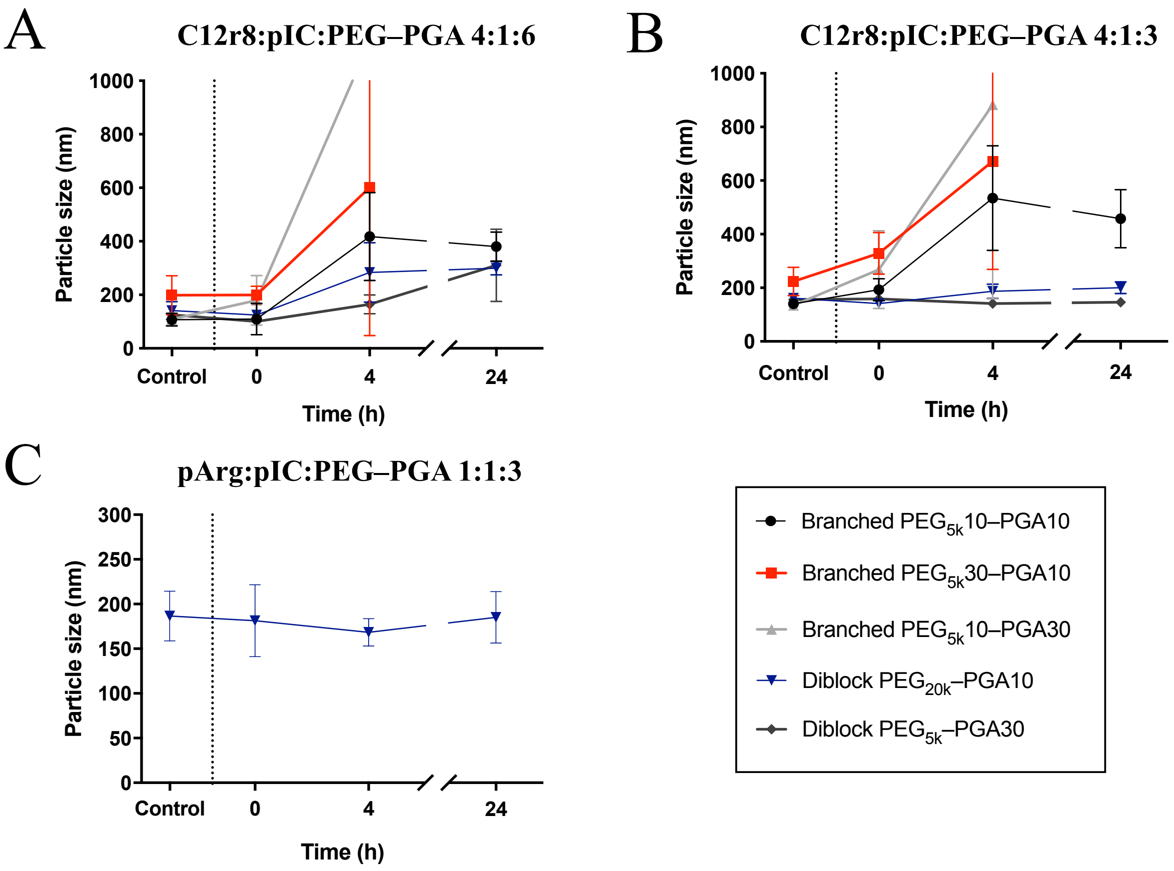

Supplementary Figure 4. Stability of the PEG–PGA enveloped nanocomplexes in cell culture media. Evolution of particle size of PEG–PGA enveloped C12r8 nanocomplexes in a weight ratio **(A)** 4:1:6 and **(B)** 4:1:3 C12r8:pIC:PEG–PGA. **(C)** Evolution of particle size of diblock PEG_{20k}–PGA10 enveloped pArg nanocomplexes in a weight ratio 1:1:3 pArg:pIC:PEG–PGA. Nanocomplexes were incubated up to 24 h in cell culture media at 37 °C, and sizes were compared with those obtained in water (control). Values represent mean ± SD (n ≥ 3). C12r8, laurate-octaarginine; pArg, poly-arginine; PEG–PGA, pegylated polyglutamic acid; PDI, polydispersity index; pIC, poly(I:C).

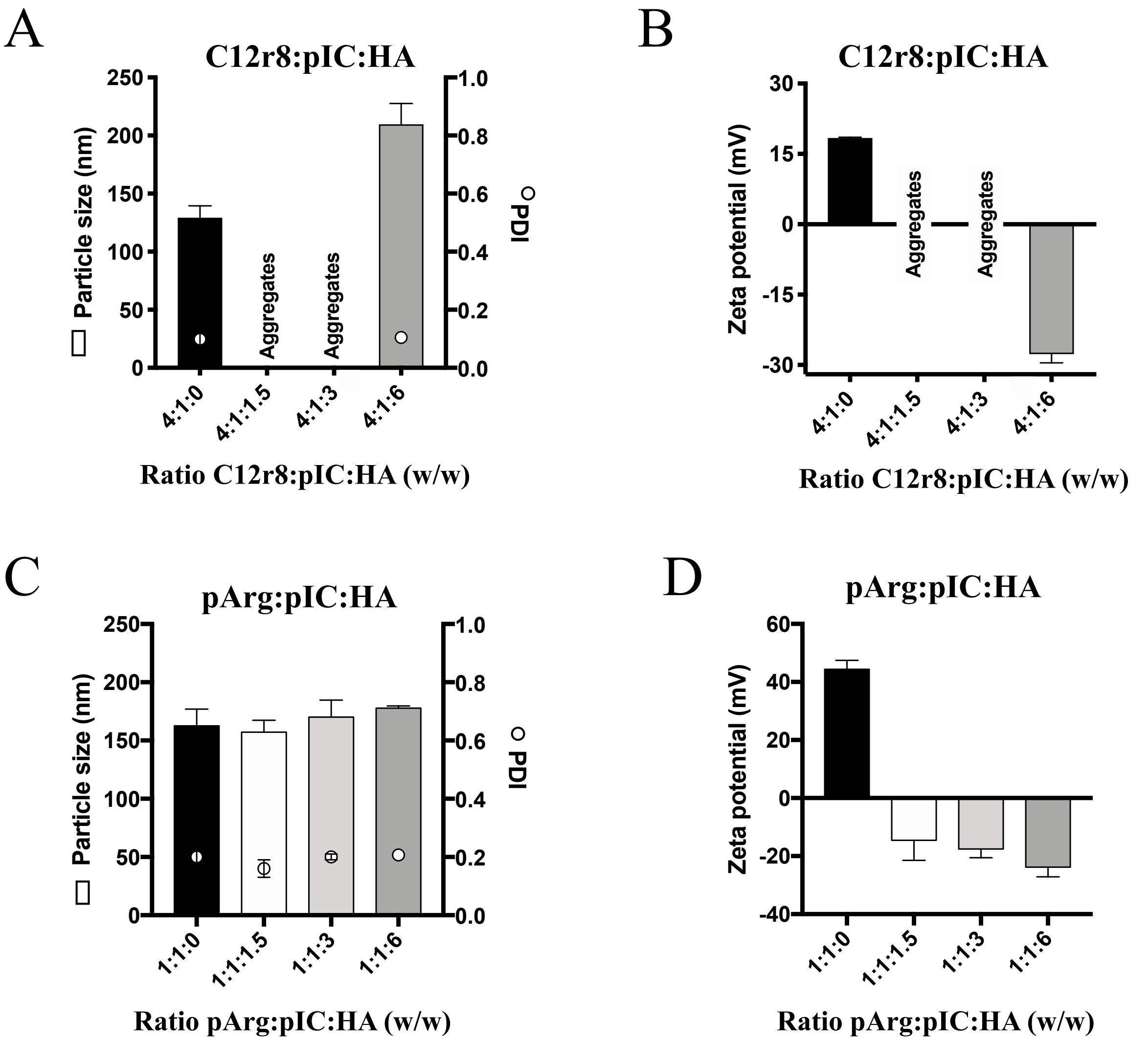

Supplementary Figure 5. Envelopment of poly(I:C)-loaded nanocomplexes with hyaluronic acid. Influence of the ratio of arginine-rich polymer:pIC:HA for the different **(A, B)** C12r8 and **(C, D)** pArg nanocomplexes. Values of **(A, C)** particle size, PDI and **(B, D)** zeta potential of the nanocomplexes obtained after adding the different ratios of hyaluronic acid. Values represent mean ± SD (n ≥ 3). C12r8, laurate-octaarginine; HA, hyaluronic acid; pArg, poly-arginine; PDI, polydispersity index; pIC, poly(I:C).

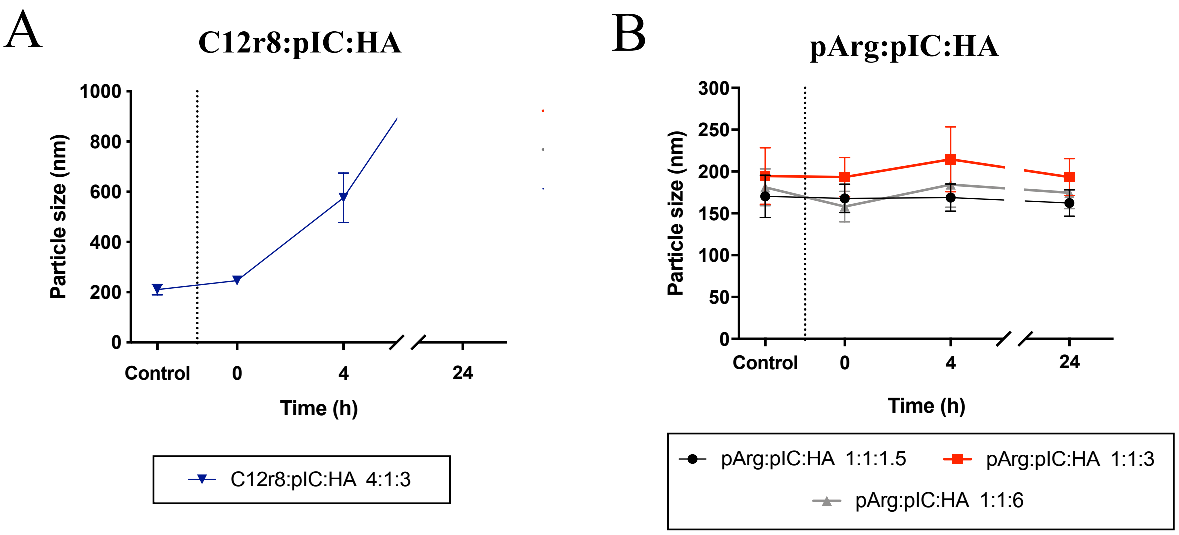

Supplementary Figure 6. Stability of the HA-enveloped nanocomplexes in cell culture media. Evolution of particle size of **(A)** C12r8-based nanocomplexes and **(B)** pArg-based nanocomplexes enveloped with different weight ratios of HA up to 24 h of incubation in cell culture media at 37 °C, in comparison with the nanocomplexes in water (control). Values represent mean ± SD (n ≥ 3). C12r8, laurate-octaarginine; HA, hyaluronic acid; pArg, poly-arginine; pIC, poly(I:C).

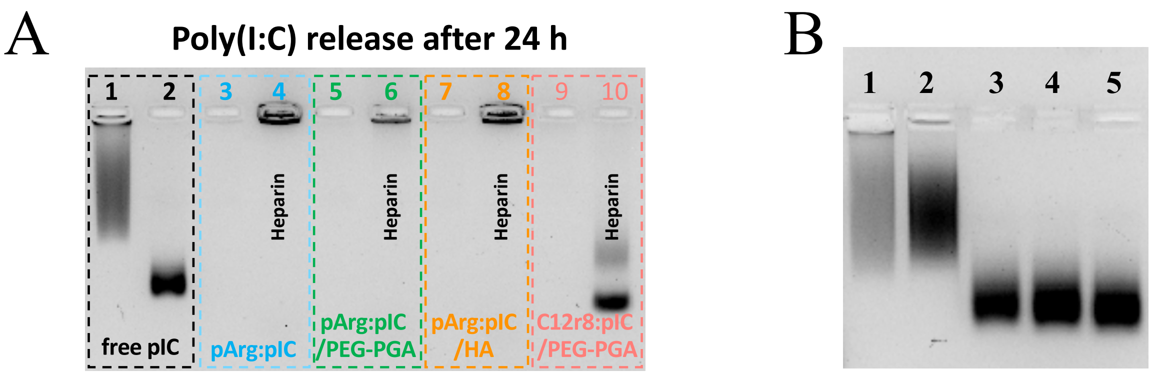

Supplementary Figure 7. Poly(I:C) release from nanocomplexes and stability in cell culture media. (A) Agarose gel retardation assay to evaluate the release and integrity of poly(I:C) after 24 h of incubation in cell culture media at 37 °C. Lanes: (1) free poly(I:C) in solution and (2) in cell culture media; (3, 5, 7, 9) are pArg:pIC, pArg:pIC/PEG–PGA, pArg:pIC/HA and C12r8:pIC/PEG–PGA nanocomplexes in cell culture media; and (4, 6, 8, 10) are the same conditions incubated with heparin**. (B)** Agarose gel retardation assay to evaluate the degradation of poly(I:C) in different conditions. Lanes: (1) free poly(I:C); (2) free poly(I:C) incubated with heparin in water for 30 min at 37 °C; (3–5) free poly(I:C) incubated in cell culture media at 37 °C for 15, 30 and 60 min, respectively. C12r8, laurate-octaarginine; HA, hyaluronic acid; pArg, poly-arginine; PEG–PGA, pegylated polyglutamic acid; PDI, polydispersity index; pIC, poly(I:C).

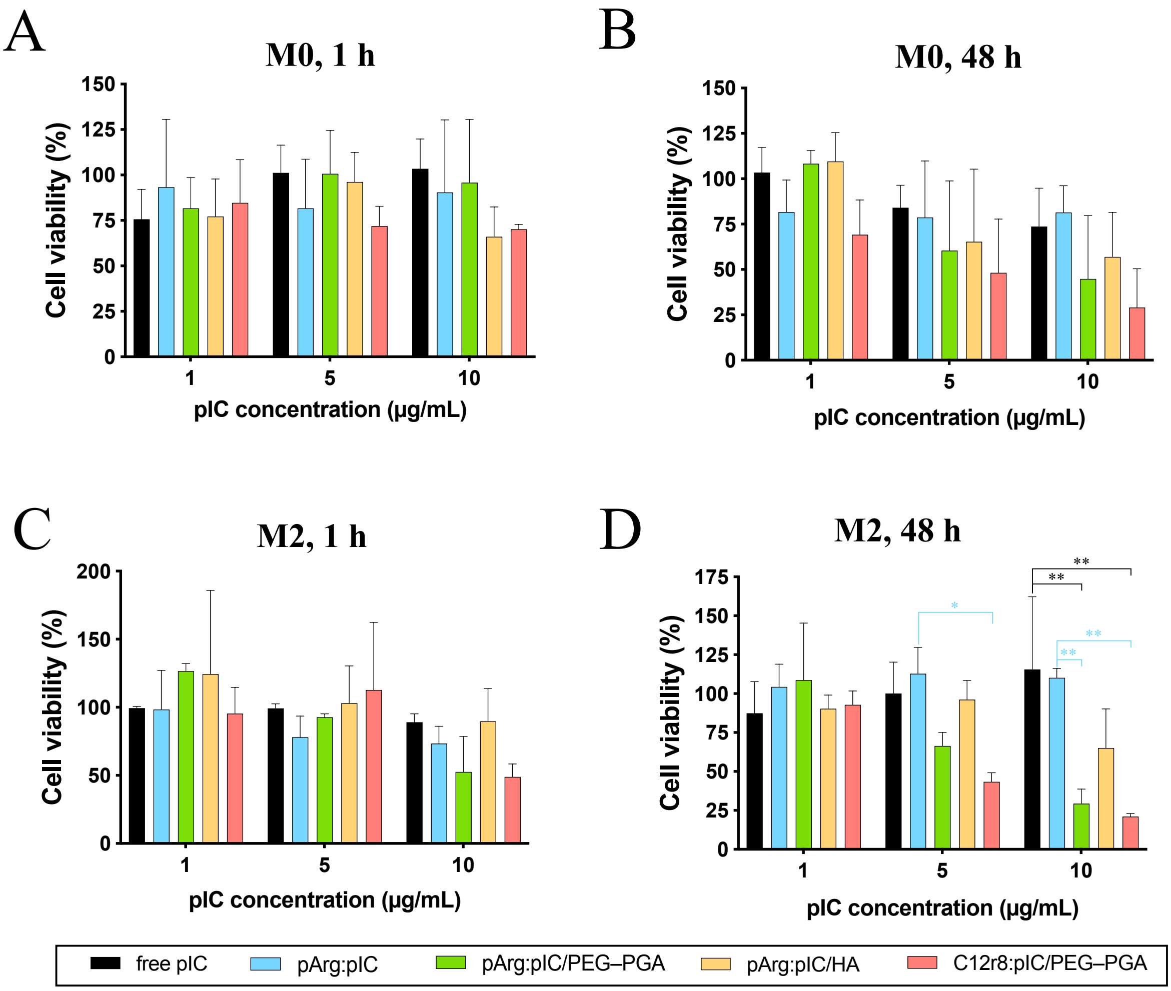

Supplementary Figure 8. Toxicity of poly(I:C)-loaded nanocomplexes towards primary human monocyte-derived macrophages. Toxicity in **(A–B)** M0 and **(C–D)** M2 macrophages after 1 and 48 h of incubation with free and nanocomplexed poly(I:C). Values represent mean ± SD (n ≥ 3). Statistical comparison was done using a two-way ANOVA followed by a Tukey's multiple comparison test, between groups. Statistically significant differences are represented as * ($p < 0.05$) and ** ($p < 0.01$). C12r8, laurate-octaarginine; HA, hyaluronic acid; pArg, poly-arginine; PEG–PGA, pegylated polyglutamic acid; pIC, poly(I:C).

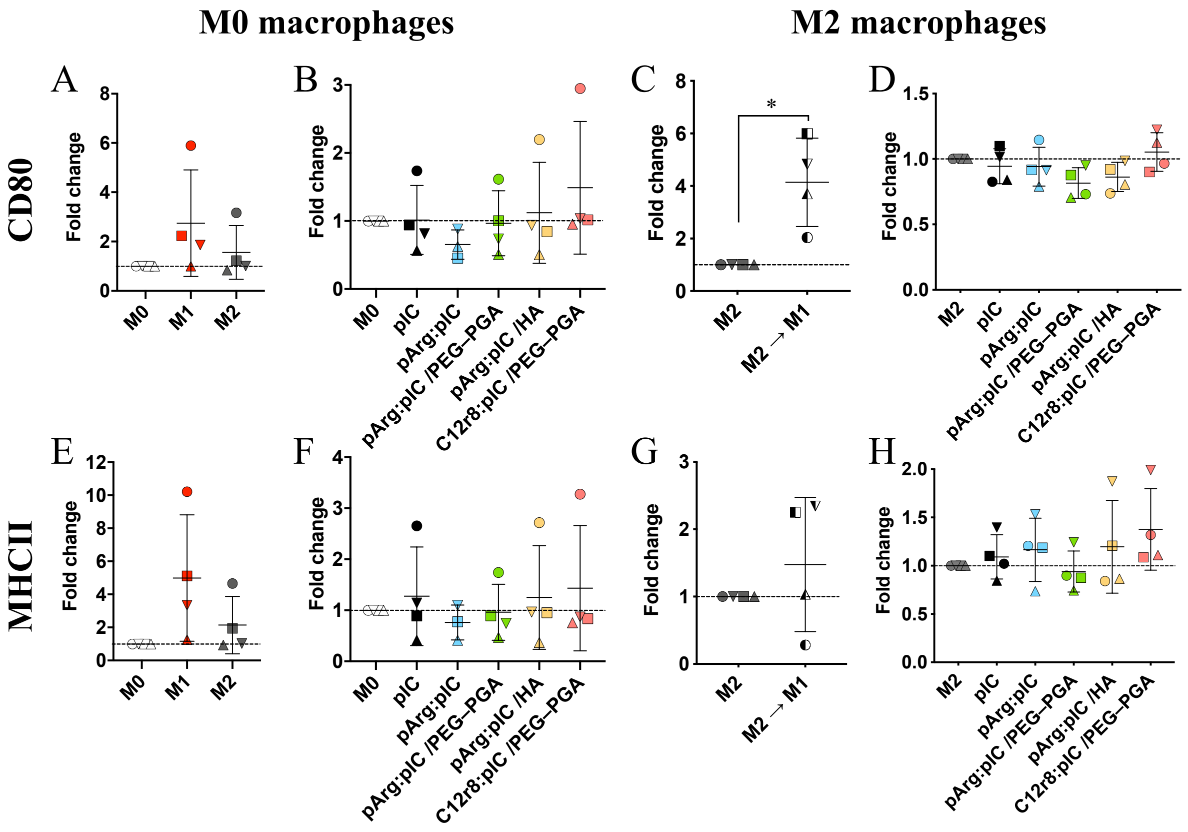

Supplementary Figure 9. Polarization of M0 and M2 macrophages after treatment with free and nanocomplexed poly(I:C) evaluated by FACS. Expression of the M1 markers **(A–D)** CD80, **(E–H)** MHCII in treated M0 and M2 macrophages, in comparison to the prototypic phenotypes. M2→ M1 represents M2 macrophages that were treated with LPS + IFN-γ for their M1 polarization. Macrophages were incubated with the treatments for 48 h, and the poly(I:C) dose used was 5 µg/mL. Each symbol shape represents a different donor. Values are shown as mean ± SD ($n \geq 3$). Statistical comparison was done using an ordinary one-way ANOVA followed by a Tukey's comparison test between groups. Statistically significant differences are represented as * ($p < 0.05$). C12r8, laurate-octaarginine; HA, hyaluronic acid; pArg, poly-arginine; PEG–PGA, pegylated polyglutamic acid; pIC, poly(I:C).

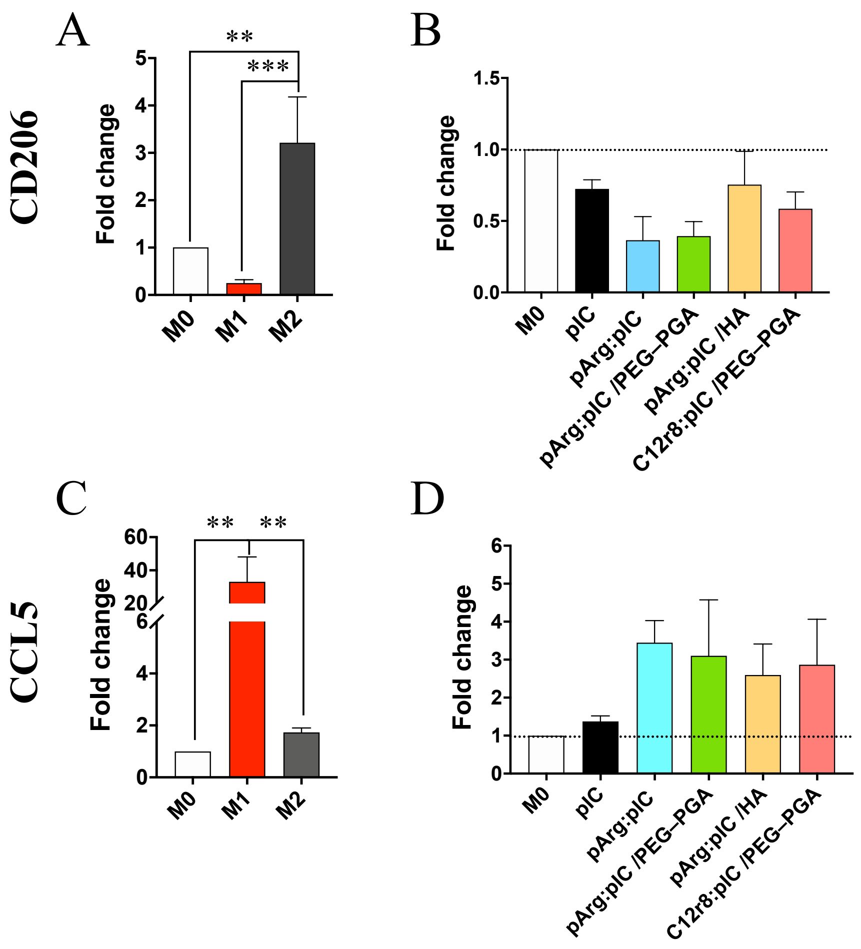

Supplementary Figure 10. mRNA production of different M1/M2 associated factors. (A–B) Fold change in the mRNA levels of *CD206* in **(A)** prototypic M1/M2 macrophages and in **(B)** M0 macrophages treated with the different nanocomplexes after 8 h of incubation. **(C–D)** Fold change in the mRNA levels of *CCL5* as chemokine in **(C)** prototypic M1/M2 macrophages and in **(D)** M0 macrophages treated with the different nanocomplexes for 8 h**.** The dose of poly(I:C) was 5 µg/mL. Values represent mean ± SD (N = 4). Statistical comparison was done using an ordinary one-way ANOVA followed by a Tukey's comparison test between groups. Statistically significant differences are represented as ** ($p < 0.01$) and *** ($p < 0.005$). C12r8, laurate-octaarginine; HA, hyaluronic acid; pArg, poly-arginine; PEG–PGA, pegylated polyglutamic acid; pIC, poly(I:C).

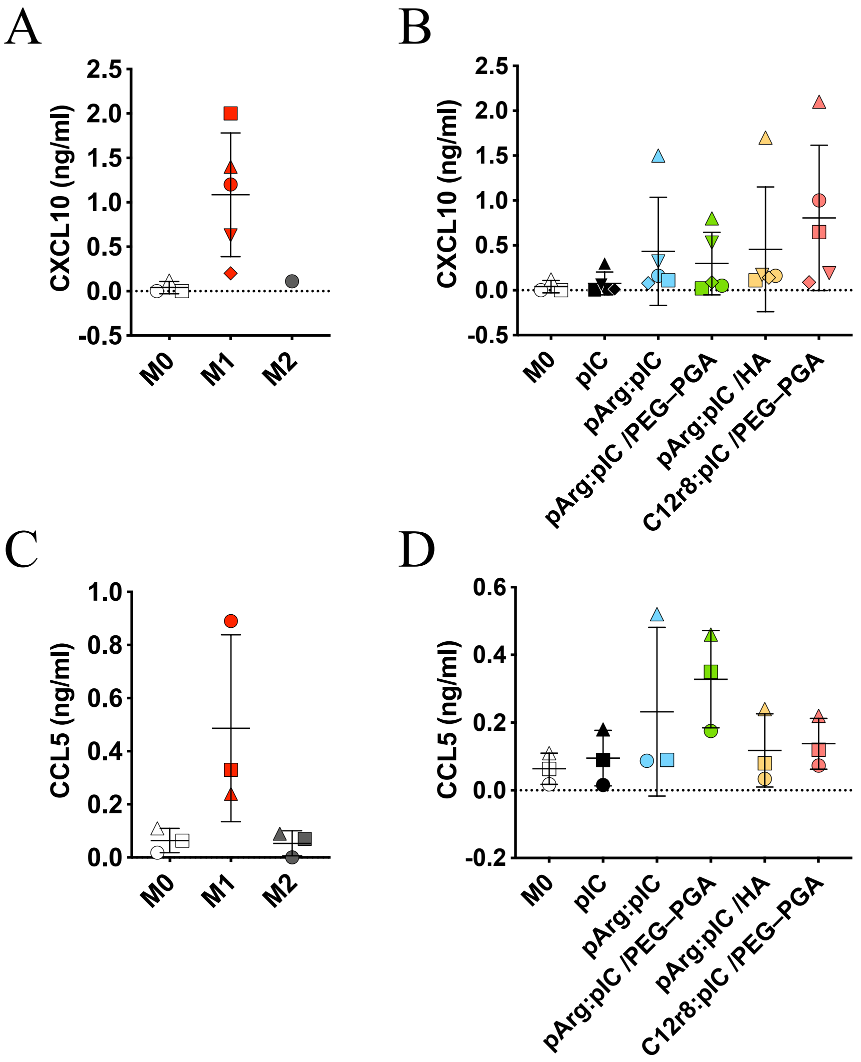

Supplementary Figure 11. Secretion of the T cell attracting chemokines CCL5 and CXCL10 upon treatment with the poly(I:C) nanocomplexes. **(A–B)** CXCL10 secretion in **(A)** M1/M2 prototypic macrophages and in **(B)** M0 macrophages treated with the different nanocomplexes after 8 h of incubation. **(C–D)** CCL5 secretion in **(C)** prototypic M1/M2 macrophages and in **(D)** M0 macrophages treated with the different nanocomplexes after 8 h of incubation. Each symbol shape represents a different donor. The dose of poly(I:C) was 5 µg/mL. Values represent mean ± SD ($n \geq 3$). Statistical comparison was done using an ordinary one-way ANOVA followed by a Tukey's comparison test groups. C12r8, laurate-octaarginine; HA, hyaluronic acid; pArg, poly-arginine; PEG–PGA, pegylated polyglutamic acid; pIC, poly(I:C).
